# Countries with delayed COVID-19 introduction – characteristics, drivers, gaps, and opportunities

**DOI:** 10.1186/s12992-021-00678-4

**Published:** 2021-03-17

**Authors:** Zheng Li, Cynthia Jones, Girum S. Ejigu, Nisha George, Amanda L. Geller, Gregory C. Chang, Alys Adamski, Ledor S. Igboh, Rebecca D. Merrill, Philip Ricks, Sara A. Mirza, Michael Lynch

**Affiliations:** Centers for Disease Control and Prevention, COVID-19 Response, 4770 Buford Highway, Atlanta, GA 30341 USA

**Keywords:** COVID-19, Pandemic, Preparedness, Global health, Surveillance, Border control measures

## Abstract

**Background:**

Three months after the first reported cases, COVID-19 had spread to nearly 90% of World Health Organization (WHO) member states and only 24 countries had not reported cases as of 30 March 2020. This analysis aimed to 1) assess characteristics, capability to detect and monitor COVID-19, and disease control measures in these 24 countries, 2) understand potential factors for the reported delayed COVID-19 introduction, and 3) identify gaps and opportunities for outbreak preparedness, particularly in low and middle-income countries (LMICs). We collected and analyzed publicly available information on country characteristics, COVID-19 testing, influenza surveillance, border measures, and preparedness activities in these countries. We also assessed the association between the temporal spread of COVID-19 in all countries with reported cases with globalization indicator and geographic location.

**Results:**

Temporal spreading of COVID-19 was strongly associated with countries’ globalization indicator and geographic location. Most of the 24 countries with delayed COVID-19 introduction were LMICs; 88% were small island or landlocked developing countries. As of 30 March 2020, only 38% of these countries reported in-country COVID-19 testing capability, and 71% reported conducting influenza surveillance during the past year. All had implemented two or more border measures, (e.g., travel restrictions and border closures) and multiple preparedness activities (e.g., national preparedness plans and school closing).

**Conclusions:**

Limited testing capacity suggests that most of the 24 delayed countries may have lacked the capability to detect and identify cases early through sentinel and case-based surveillance. Low global connectedness, geographic isolation, and border measures were common among these countries and may have contributed to the delayed introduction of COVID-19 into these countries. This paper contributes to identifying opportunities for pandemic preparedness, such as increasing disease detection, surveillance, and international collaborations. As the global situation continues to evolve, it is essential for countries to improve and prioritize their capacities to rapidly prevent, detect, and respond, not only for COVID-19, but also for future outbreaks.

**Supplementary Information:**

The online version contains supplementary material available at 10.1186/s12992-021-00678-4.

## Background

**Fig. 1:**
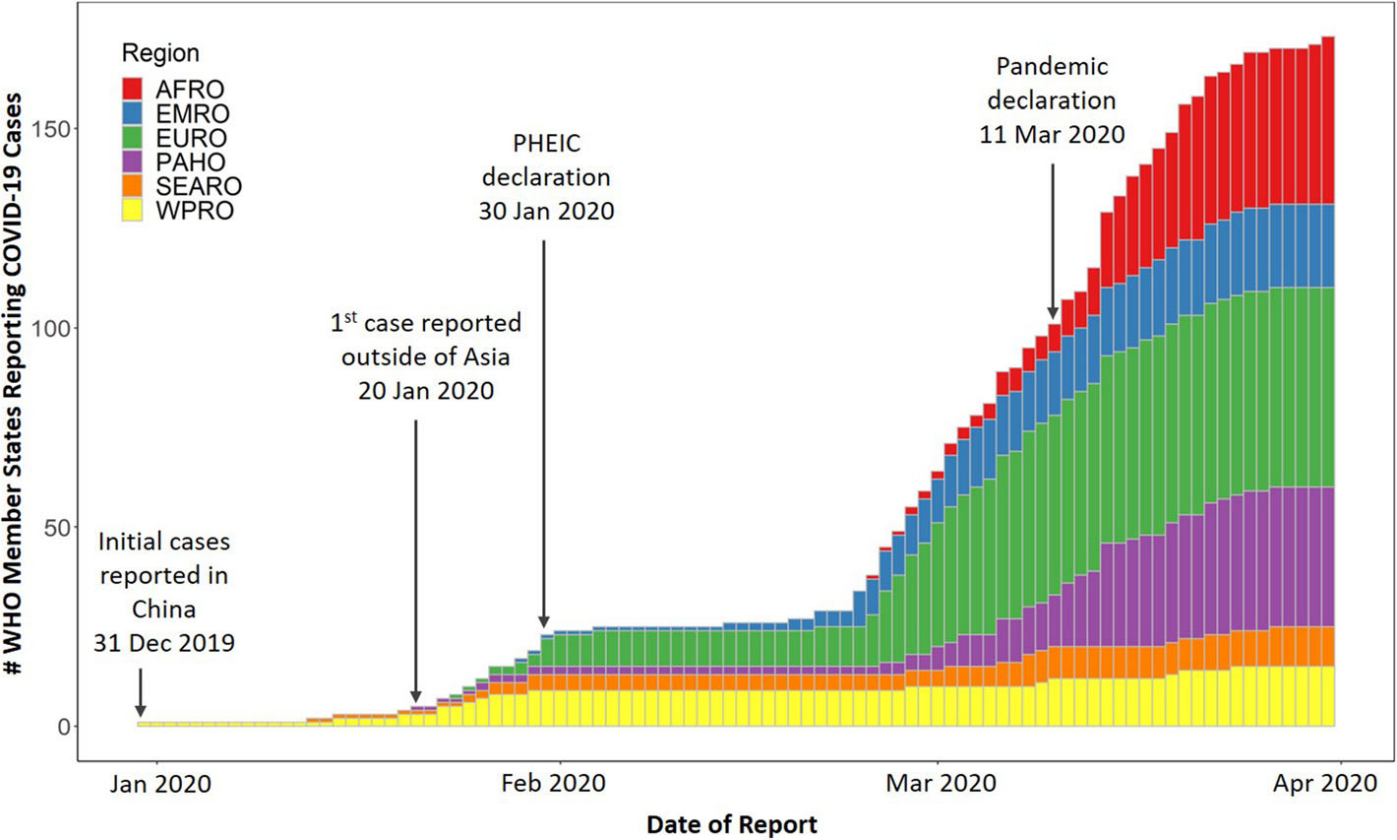
Daily number of countries with reported cases by World Health Organization regions, 31 Dec 2019–30 Mar 2020. (PHEIC: Public Health Emergency of International Concern)

Infectious diseases recognize no borders and can easily spread into new geographic areas. Three months after the first reported cases of coronavirus disease 2019 (COVID-19) [1], the infection had spread rapidly throughout the world (Fig. 1). Confirmed cases had been reported in 88% of the 195 WHO member states (hereafter referred to as countries); only 24 countries had not reported cases to WHO as of 30 March 2020, based on WHO COVID-19 Situation Reports [2]. Many factors can contribute to the emergence and transmission of infectious diseases on a global scale [3, 4]. COVID-19 has an average basic reproductive number of 3.38 ± 1.4 (range 1.9–6.5) [5], and an average incubation period of 5–6 days [6], with infectiousness starting approximately 2–3 days before symptom onset [7, 8]. In addition, asymptomatic transmission [9] makes tracking more complicated and difficult. These attributes, paired with complex human connectivity between countries, especially with regards to international travel, significantly affected the spread of the outbreak. When facing rapidly developing outbreaks, countries can proactively take public health measures to delay introduction and interrupt disease transmission [10]. Public health surveillance and testing capabilities are crucial to detect the introduction and spread of disease and to contain novel emerging infections, like COVID-19, especially during an outbreak’s early stages [11].

Noting the speed, scale, and intensity of the COVID-19 pandemic and the devastation to countries, it is important to understand factors that can affect the spread of the virus and to subsequently identify potential gaps and opportunities for outbreak preparedness. This is especially important for low and middle-income countries (LMIC) with limited economic and healthcare resources [12, 13]. We conducted a focused analysis on the “final” 24 countries that had not reported COVID-19 cases to WHO as of 30 March 2020 based on a variety of factors related to the disease prevention, detection, and preparedness. Our objectives were to 1) assess country characteristics, capability and capacity to detect the introduction and monitor spread of COVID-19, and the disease control measures implemented in these 24 countries, 2) identify characteristics and factors that may be related to delayed importation of COVID-19, and 3) identify gaps and opportunities for outbreak preparedness to combat COVID-19 and inform future outbreak preparedness, particularly in countries with limited resources.

## Methods

We identified the 24 countries that had not reported COVID-19 cases to WHO as of 30 March 2020 based on data released on WHO COVID-19 Situation Reports [2]. We collected publicly available information on each of the 24 countries that may be related to the objectives of this study. Multiple information and data sources were used, including official websites and social media platforms (e.g., Facebook and Twitter) of country-specific entities (e.g., ministries of health and other government agencies), as well as international organizations (e.g., WHO and other United Nations agencies). We organized the data into several broad categories (Table 1), including country characteristics and COVID-19-related information. Country characteristics included economic development status [14], vulnerable country designation [15], health security and capability indicator (as measured by Global Health Security [GHS] Index) [16], globalization indicator (as measured by Global Connectedness Index [GCI]) [17], annual inbound foreign visitors [18], population [19], and healthcare indicators [19]. COVID-19-related information included in-country testing capability and capacity for detecting SARS-CoV-2 (the virus responsible for COVID-19), existing influenza surveillances [20], border measures (travel advisory, border closure, traveler screening and quarantine), and preparedness activities, such as national COVID-19 preparedness plan, strategy or task force, healthcare worker trainings, acquisition of personal protective equipment (PPE), and mitigation strategies (e.g. mass gathering restrictions, and school and business closures). Accession dates ranged from 30 March 2020 to 10 April 2020. The entire list of sources is provided in the Supplemental Materials (Tables S1-S4).

**Table 1:** Variables included in the analysis, definitions, and sources

Variable	Definition	Source
**Country characteristics**		
Level of development	Level of development as measured by per capita gross national income: high-income, upper-middle-income, lower-middle-income and low-income	UN World Economic Situation and Prospects (2019)
Vulnerability classifications	Vulnerable country designations by the United Nations: Least Developed Countries, Landlocked Developing Countries, and Small Island Developing States	UN Office of the High Representative for the Least Developed Countries, Landlocked Developing Countries and the Small Island Developing States
Inbound visitor arrival (per capita population)	Calculated ratio of inbound visitor arrivals at national borders (non-resident visitors, including overnight and same-day visitors, tourists and excursionists) to country population	UN World Tourism Organization (2017–2018); WHO
Global connectedness index (GCI)	An index measures globalization based on trade, capital, information, and people flows; ranking is available on 169 countries and areas: higher rank indicates less global connectedness	Altman et al., DHL Global Connectedness Index 2018 - The State of Globalization in a Fragile World (2019)
**Health security and healthcare indicators**		
Global health security index	An index on comprehensive assessment of global health security capabilities in 195 countries that make up the States Parties to the International Health Regulations; ranking on Overall and on Detection are presented (out of 195): higher rank indicates lower health security capabilities	Global Health Security Index (2019)
# Doctor (per 10 k)	Number of doctors per 10,000 population	WHO Global Health Observatory
# Nurse & midwives (per 10 k)	Number of nurses and midwives per 10,000 population	
**COVID-19 testing and influenza surveillance**		
In-country COVID-19 testing (if yes, capacity)	If reporting available test kits or COVID-19-capable laboratories, then “Yes”. If a country has test kits but explicitly states no lab, then “No”. If yes, brief description of testing capacity	Official websites and social media channels of country Ministries of Health and other governments, and U.S. Embassies
Export COVID-19 testing	If no in-country testing capability, report that samples had been sent to another country for testing or a plan to export samples for testing	
Influenza surveillance (if yes, type)	Reporting influenza surveillance information to WHO Global Influenza Programme from May 2019 and April 2020. If yes, reported laboratory (Lab) and/or epidemiology (ILI: influenza-like illness, SARI: severe acute respiratory infections) surveillances	WHO Global Influenza Programme (2019–2020)
**Border control measures**	*Yes/No/Unknown*	
Travel restrictions	Any international flight suspensions, restricted air/land/sea border crossing activities, or suggestions to postpone travel outside of the country	Official websites and social media channels of country Ministries of Health and other governments, and U.S. Embassies
Border closures	Suspension of air/land/sea-based points of entry. Island nations suspending cruise ship docking	
Screening at points of entry	Symptom checks screening (e.g. temperature checks), travel history taken, and/or requirement of certification of COVID-19 free	
Traveler quarantine	14-day isolation of travelers entering the country, mandatory or advised, self-isolation or government quarantine at a facility, or initial self-quarantine in country of origin or other disease-free area	
**COVID-19 preparedness activities**	*Yes/No/Unknown*	
COVID-19 preparedness plan or strategy	Mention of a COVID-19 task force, a plan or a strategy for preparing the country for the arrival of COVID-19, either by the country government or through collaboration with WHO, UN, or another country	Official websites and social media channels of country Ministries of Health and other governments, and U.S. Embassies
Mass gathering restrictions	Instructions and/or announcements to limit gatherings to a specified number of people or less; mandatory or advised/encouraged	
School closures	Extension of school holidays or complete school cancellation	
Business closures	Complete closure of some businesses or all non-essential businesses	
Funding for COVID-19 activities	Financial resources released by the government or received from an external partner designated for COVID-19 preparedness	
Healthcare worker training	COVID-19 trainings or refreshers for healthcare workers	
Availability of PPE	Acquisition of a supply of masks, gloves, gowns, respirators, or face shields that’ll be available to health workers.	
Quarantine/isolation facilities	Designated locations for travelers or other exposed persons to quarantine or self-isolate for 14 days	

We standardized the variables in the categories of testing capabilities, mitigation measures, and preparedness activities (Table 1). Variables were summarized as “Yes” or “No” if relevant sources indicated the presence or absence of a variable, and as “Unknown” if we were unable to find information from relevant sources. Due to the rapid evolution of preventive measures and the overall objective of this analysis, we only recorded the initial implementation date and the most recent updates as of 30 March 2020 and did not include all specific iterations of the measures.

To study the potential effects of global connectedness and geographic factor on the spread of COVID-19, we conducted analyses on the temporal spreading of COVID-19 among all countries with reported cases with their GCI rank, a globalization indicator reflecting movement of people, trade, information, and capital [17]. We first determined the number of days elapsed between the initial reporting date to WHO (31 December 2019) and the date of the index case for all individual countries with confirmed cases as reported by WHO. We then applied Spearman correlation analysis and multiple linear regression to assess the relationship between number of days to reported index case against the GCI rank in 166 countries with available GCI scores and the country’s geographic location (continent). In this analysis, we included three countries with available GCI ranks that reported cases between 30 March and the time of the analysis on 10 April 2020 (Botswana, Sierra Leone, and Yemen). The analyses were conducted using SAS 9.4.

## Results

**Fig. 2:**
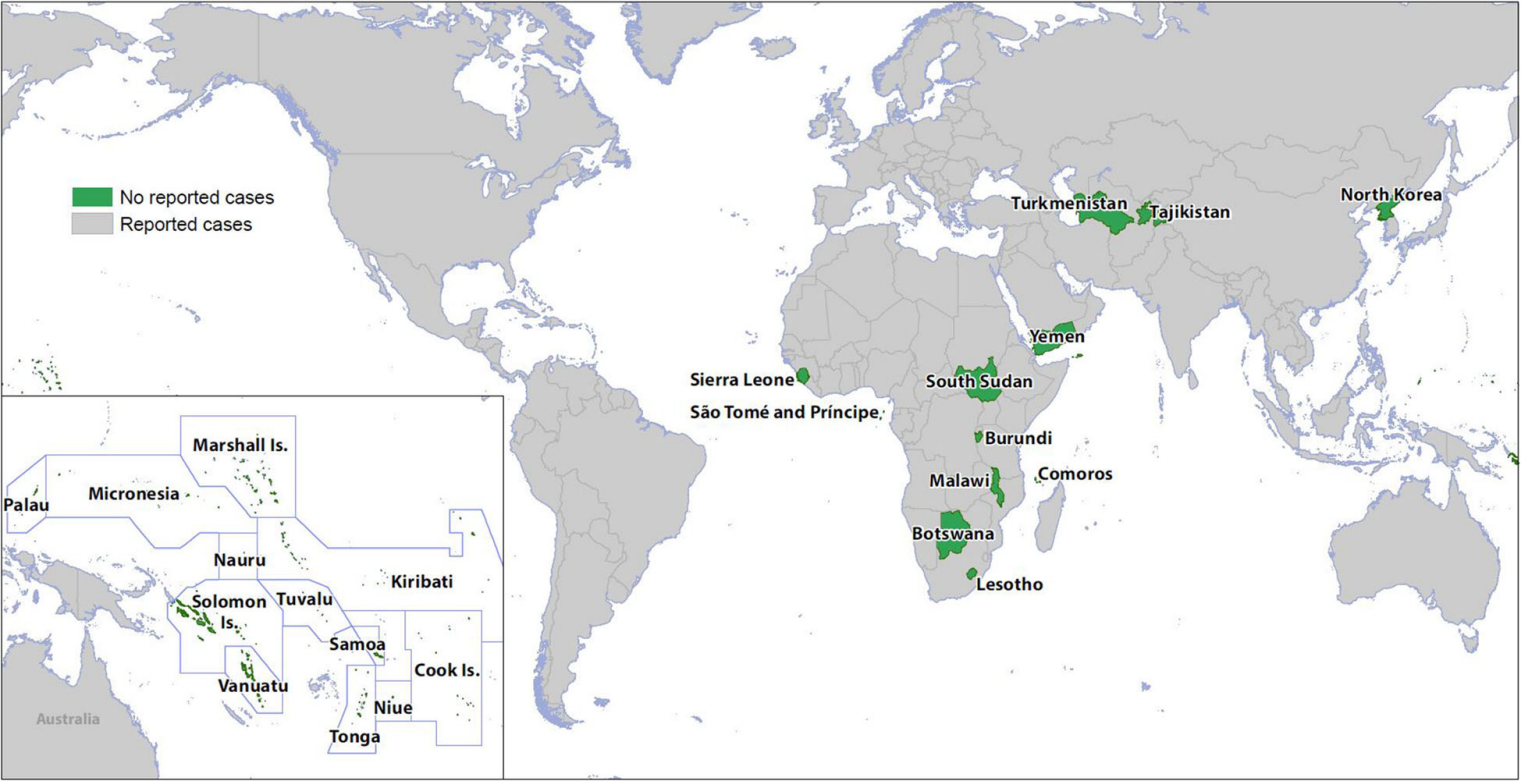
Map of the 24 countries with no reported cases as of 30 March 2020

Among the 24 countries that had not reported a COVID-19 case as of 30 March 2020, 12 (50%) were in Western Pacific (WPRO), 8 (30%) in Africa, 2 (8%) in Europe, and 1 each in Eastern Mediterranean (EMRO) and South-east Asia (SEARO) (Fig. 2), collectively representing a total of 107 million people or roughly 1.4% of the global population. Of the 24 countries, 14 (58%) are small island developing countries, i.e., 12 countries in WPRO and 2 in Africa, 7 (29%) are landlocked developing countries, and 7 (29%) are classified as the least developed countries (Table 2) [15]. Except for the two European countries (Tajikistan and Turkmenistan) that are categorized as economies in transition, all remaining countries are developing countries, most of which are categorized as lower-middle income or low-income. Moreover, most of the African countries are classified as heavily indebted poor, least developed, or both (Table 2) [14, 19].

**Table 2:** Selected characteristics, global health security index, and healthcare system indicators in the 24 countries with no reported COVID-19 cases as of 30 March 2020

Country	Country characteristics				Health security and healthcare indicators			
	Level of development	Vulnerability classification	Global connectedness index (Rank of 169)	Inbound visitor arrivals/Popl. (Year)	Global health security index (rank of 195)		Number per 10 K population	
					Overall	Detection	Doctors	Nurse/midwives
**AFRO**								
Botswana	Upper-middle-income	Landlocked developing	147	0.79 (2017)	139	133	5.3	54
Burundi	Low-income	Landlocked developing & Least developed	NA	0.03 (2017)	177	175	1.0	8.5
Comoros	Low-middle-income	Small island developing & Least developed	161	0.05 (2018)	160	148	2.7	6.3
Lesotho	Low-middle-income	Landlocked developing & Least developed	145	0.53 (2018)	144	160	0.69	32.6
Malawi	Low-income	Landlocked developing & Least developed	NA	0.05 (2018)	154	146	0.36	4.4
Sao Tome and Principe	Low-middle-income	Small island developing & Least developed	NA	0.17 (2018)	192	194	0.53	19.2
Sierra Leone	Low-income	Least developed	99	0.01 (2018)	92	72	0.25	2.2
South Sudan	Low-income	Landlocked developing & Least developed	NA	NA	180	166	0.15	12.2
**EMRO**								
Yemen	Low-income	Least developed	165	NA	190	179	5.3	7.9
**EURO**								
Tajikistan	Low-income	Landlocked developing	159	0.12 (2018)	130	144	21	47.5
Turkmenistan	Upper-middle-income	Landlocked developing	NA	NA	135	101	22.2	44.3
**SEARO**								
North Korea	Low-income	NA	NA	NA	193	185	36.8	44.4
**WPRO**								
Cook Islands	NA	Small island developing	NA	9.94 (2018)	185	180	14.1	67.4
Kiribati	Low-middle-income	Small island developing	166	0.08 (2018)	189	189	2.0	38.3
Marshall Islands	NA	Small island developing	94	0.13 (2018)	191	189	4.2	33.4
Micronesia	NA	Small island developing	NA	0.18 (2018)	124	171	1.9	20.4
Nauru	NA	Small island developing	NA	NA	182	189	14.0	76.6
Niue	NA	Small island developing	NA	6.00 (2017)	184	189	18.7	125
Palau	NA	Small island developing	117	4.82 (2018)	179	180	14.2	72.6
Samoa	Upper-middle-income	Small island developing	148	0.88 (2018)	162	173	3.4	24.9
Solomon Islands	Low-middle-income	Small island developing	129	0.05 (2018)	183	182	1.9	21.6
Tonga	NA	Small island developing	107	0.72 (2018)	171	167	5.4	41.6
Tuvalu	NA	Small island developing	NA	0.27 (2018)	181	182	9.1	42.6
Vanuatu	Low-middle-income	Small island developing	154	1.30 (2018)	165	167	1.7	14.2

Most of the 24 countries have limited healthcare resources (Table 2), with a median of 3.8 doctors (range 0.15–36.8) and 33 nurses and midwives (range 2.2–125) per 10,000 population; as a comparison, the U.S. has 26.1 doctors and 145.5 nurses and midwives per 10,000 population [19]. Based on Global Health Security (GHS) index, the average ranking of 19 countries with available information is 166 out of 195 (median 178, range 92–193) for overall indicator, indicating their low capability on health security [16].

Among the 19 countries with available data on annual number of arrivals of non-resident visitors (overnight visitors, tourists, same-day visitors, or excursionists) at national borders [18], the per capita inbound visitor arrivals ranged from 0.01 in Sierra Leone to 9.94 in Cook Islands, with a median of 0.18 (Table 2). The WPRO countries had higher per capita inbound visitors (median 0.72, range 0.05–9.94, *n* = 11) compared to the countries outside of WPRO (median 0.08, range 0.01–0.79, *n* = 8) and the U. S (0.53).

**Fig. 3:**
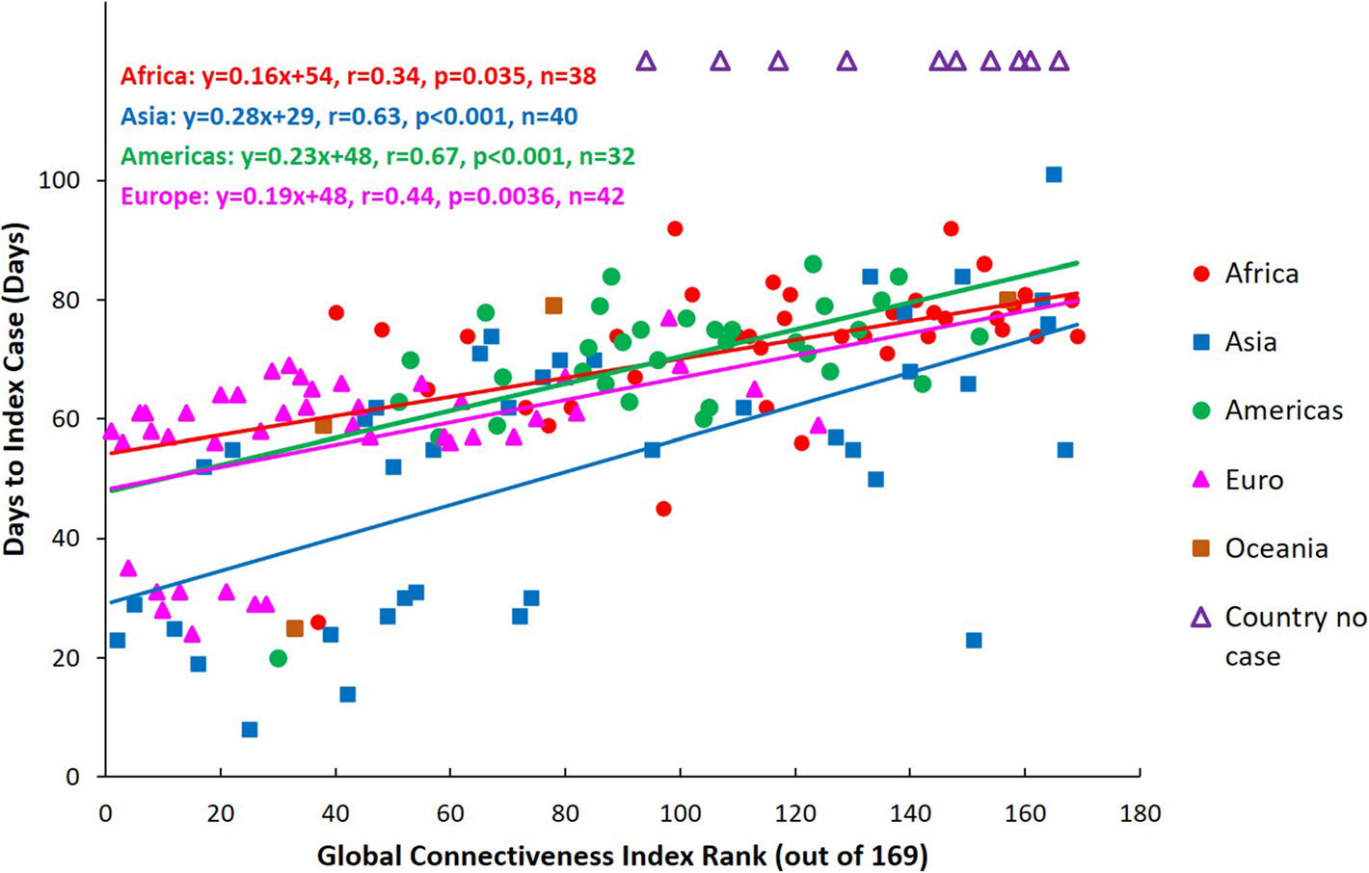
Linear correlation of Global Connectedness Index rank and days since the initial reporting of COVID (31 December 2019) to the index case in 166 countries with reported COVID-19 cases, stratified by continent

For the 13 countries with available Global Connective Index (GCI), a broad globalization indicator [17], the median global rank was 147 out of 169 (range 94–166), indicating these countries were among the lowest in global connectedness. We found strong and significant correlations between days elapsed to index case reporting and countries’ GCI rank, both for all countries combined (*r* = 0.66, *p* < 0.0001, *n* = 155), and when stratified by continents (Fig. 3), i.e. African (*r* = 0.34, *p* = 0.035, *n* = 38), Asia (*r* = 0.63, *p* < 0.0001, *n* = 40), Europe (*r* = 0.44, *p* = 0.0036, *n* = 42), North America (*r* = 0.74, *p* < 0.0001, *n* = 21), and South America (*r* = 0.61, *p* = 0.046, *n* = 11). Oceania also exhibited a positive though non-significant correlation (*r* = 0.72, *p* = 0.28, *n* = 4) with only four countries with GCI reporting cases. Further, as indicated in Fig. 3, although Asian countries tended to have shorter elapsed time to COVID-19 introduction than other continents, the geographic effect diminished with decreased global connectedness (increased GCI). Multiple linear regression analysis on all 155 countries reporting cases and with GCI further confirmed that the temporal spreading was significantly associated with the GCI rank (beta with 95% CI: 0.23 [0.19, 0.27], *p* < 0.0001), location variable using Asia as the reference (14 [9, 18], *p* < 0.0001), with an intercept of 19 (10, 29) and adjust *R*^2^ = 0.47 for the model.

SARS-CoV-2 testing capability, influenza surveillance, border control measures, and COVID-19 preparedness activities are summarized in Table 3 (detailed information and sources are provided in Supplemental Materials Tables S1-S4). As of 30 March 2020, 9 (38%) of the countries had reported in-country capability to test for SARS-CoV-2. Among them, four reported information on testing capacity for SARS-CoV-2, either on total number of available testing kits (20,000 or more total kits for Malawi and South Sudan) or on daily testing throughput (Botswana 500 tests/day, Sierra Leone 35 tests/million people/day). Among the 15 countries with no in-country testing, 10 either had reported exporting samples to other countries for testing or had planned to do so upon the arrival of a suspected case.

**Table 3:** COVID-19 testing capacity, influenza surveillance, border control measures, and preparedness activities in the 24 countries with no reported cases as of 30 March 2020

Country	Testing and surveillance			Border control measures				COVID-19 preparedness activities							
	In-country COVID-19 testing (if yes, capacity)	Export COVID-19 testing	Flu surveillance (if yes, type^**a**^)	Travel restriction	Border closure	Entry screening	Traveler quarantine	Preparedness plan	Mass gathering restriction	School closure	Business closure	COVID-19 funding	Health worker training	PPE^**b**^	Quarantine isolation facility
**AFRO**															
Botswana	Yes (500/day)	–	No	Yes	Yes	Yes	Yes	Yes	Yes	Yes	Yes	Yes	Yes	Yes	Unknown
Burundi	Yes (Unknown)	–	No	Yes	Unknown	Yes	Yes	Unknown	Unknown	No	Unknown	Yes	Unknown	Yes	Yes
Comoros	No	Unknown	No	Yes	Yes	Yes	Yes	Yes	Yes	Yes	Unknown	Yes	Yes	Yes	Yes
Lesotho	No	Yes	No	Yes	Yes	Yes	Yes	Yes	Yes	Yes	Yes	Yes	Yes	Yes	Yes
Malawi	Yes (> 20 K total tests)	–	No	Yes	Unknown	Yes	Yes	Yes	Yes	Yes	Yes	Yes	Yes	Yes	Unknown
Sao Tome and Principe	No	Yes	No	Yes	Yes	Yes	Yes	Yes	Yes	Yes	Yes	Yes	Unknown	Yes	Unknown
Sierra Leone	Yes (35/mil people/day)	–	Yes (Lab, ILI, SARI)	Yes	Yes	Yes	Yes	Yes	Yes	Yes	Yes	Yes	Yes	Unknown	Yes
South Sudan	Yes (20 K total tests)	–	Yes (Lab, ILI, SARI)	Yes	Yes	Yes	Yes	Yes	Yes	Yes	Yes	Yes	Yes	Yes	Yes
**EMRO**															
Yemen	Yes (Thousands)	–	Yes (Lab)	Yes	Yes	Yes	Yes	Yes	No	Yes	Unknown	Yes	Yes	Yes	Unknown
**EURO**															
Tajikistan	Yes (Unknown)	–	Yes (Lab, ILI, SARI)	Yes	Unknown	Unknown	Yes	Unknown	Unknown	Unknown	Unknown	Yes	Unknown	Unknown	Yes
Turkmenistan	Yes (Unknown)	–	Yes (Lab, ILI, SARI)	Yes	Yes	Yes	Yes	Yes	Unknown	Unknown	Unknown	Yes	Unknown	Yes	Yes
**SEARO**															
North Korea	Yes (Unknown)	–	Yes (Lab, ILI, SARI)	Unknown	Unknown	Yes	Yes	Yes	Yes	Yes	Unknown	Unknown	Unknown	Yes	Yes
**WPRO**															
Cook Islands	No	Yes	Yes (ILI)	Yes	Yes	Unknown	Yes	Yes	Yes	Yes	Yes	Yes	Yes	Yes	Unknown
Kiribati	No	Yes	Yes (ILI)	Unknown	No	Yes	Yes	Yes	No	Yes	Unknown	Unknown	Unknown	Unknown	Unknown
Marshall Islands	No	Yes	Yes (ILI)	Yes	Yes	Yes	Unknown	Yes	Unknown	Yes	Unknown	Yes	Yes	Yes	Yes
Micronesia	No	Yes	Yes (ILI)	Yes	Unknown	Yes	Yes	Yes	Unknown	Unknown	Unknown	Yes	Unknown	Unknown	Yes
Nauru	No	Yes	No	Yes	No	Yes	Yes	Yes	No	No	No	Yes	Unknown	Yes	Yes
Niue	Unknown	Unknown	Yes (ILI)	Yes	No	Unknown	Yes	Yes	Unknown	Yes	Unknown	Yes	Unknown	Unknown	Unknown
Palau	No	Yes	Yes (ILI)	Yes	No	Yes	Yes	Yes	Yes	Yes	Unknown	Yes	Unknown	Unknown	Unknown
Samoa	No	Yes	Yes (ILI)	Yes	Yes	Yes	Yes	Yes	Yes	Yes	Yes	Yes	Unknown	Yes	Unknown
Solomon Islands	No	Yes	Yes (ILI)	Yes	Yes	Unknown	Yes	Yes	Unknown	Unknown	Yes	Yes	Unknown	Yes	Yes
Tonga	Unknown	Unknown	Yes (ILI)	Yes	Yes	Yes	Yes	Yes	Yes	Yes	Yes	Unknown	Unknown	Unknown	Unknown
Tuvalu	Unknown	Unknown	Yes (ILI)	Yes	Unknown	Yes	Unknown	Yes	Yes	Unknown	Unknown	Unknown	Unknown	Unknown	Unknown
Vanuatu	No	Unknown	Yes (ILI)	Yes	Yes	Unknown	Yes	Yes	Yes	Yes	No	Yes	Yes	Yes	Yes

^a^*Lab* laboratory confirmed influenza, *ILI* influenza-like illness, *SARI* severe acute respiratory infections

^b^*PPE* personal protective equipment

Based on WHO Global Influenza Programme [20], 17 (71%) of the countries had reported influenza surveillance data from May 2019 to April 2020 (Table 3). Of the 17 countries, Yemen only reported virologic laboratory influenza data; 11 only reported influenza-like illness (ILI) data; and 5 reported laboratory-confirmed influenza, surveillance on ILI, and severe acute respiratory infections (SARI). The 12 WPRO countries participate in the Pacific Public Health Surveillance Network (PPHSN) [21] that tracks ILI, acute fever and rash, diarrhea and prolonged fever using the Pacific Syndromic Surveillance System [22].

**Fig. 4:**
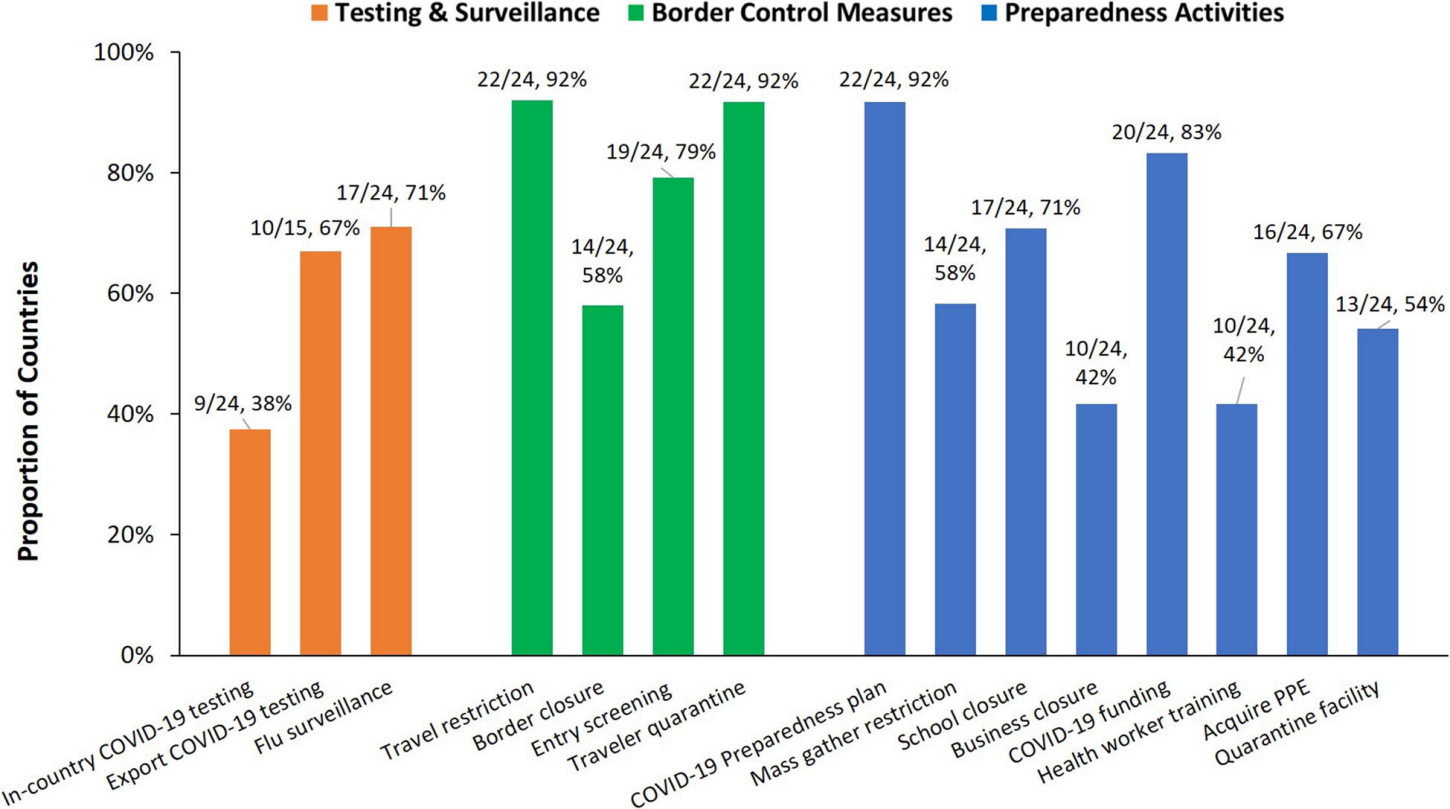
COVID-19 testing capability, existing influenza surveillances, border control measures, and preparedness activities in the 24 countries with no reported cases as of 30 March 2020. The denominator for exporting COVID-19 testing is the 15 countries with no in-county testing capability

As of 30 March 2020, all 24 countries had implemented multiple border control measures to prevent the introduction of COVID-19 into their country (Table 3, Supplemental Materials Tables S2 and S3), including travel restrictions (92%, 22/24, 2 unknown), closing air, land and/or sea borders (58%, 14/24, with exceptions such as essential, emergency, or citizen crossings), screening at points of entry (79%, 19/24), or quarantines for individuals entering the country (92%, 22/24) (Fig. 4). All countries had also begun multiple preparedness activities (Tables 3 and S4). Of countries with available information, all (22/24, 2 unknown) announced national preparedness strategies, plans, or task forces created either by the government or through collaboration with WHO, other UN agencies, or other countries; all (20/24, 4 unknown) had allocated funding for COVID-19. Furthermore, despite having no confirmed cases, 17 countries had pre-emptively closed schools and or non-essential businesses; 14 had implemented mass gathering restrictions. Ten countries announced training healthcare workers for COVID-19 response, 16 either acquired or were acquiring additional PPE, and 13 had designated locations for travelers or other exposed persons to quarantine or isolate.

## Discussion

In recent years, multiple efforts have been made to predict and prevent the next pandemic and promote countries’ pandemic preparedness [23, 24]. The Global Health Security (GHS) Index, a comprehensive assessment of health security and related capabilities based on six categories (prevention, detection and reporting, response, health system, internal norm, and risk environment), scored countries to spur measurable changes in national health security and improve international capability to prepare for disease epidemic and pandemic outbreaks [16]. COVID-19, a highly contagious emerging infectious disease, rapidly evolved into a global pandemic two and a half months after its first reported case, increasing drastically both in the number of cases and deaths and in the number of countries reporting infections.

International travel is known to increase the risk of imported cases into countries and studies had assessed COVID-19 importation risk using international air travel data [10, 25]. However, other international movements (e.g., by land, sea, non-commercial air, goods) also contribute to importation risk. We used GCI, a broad global connectedness indicator, in our analysis and found that increased global connectedness was strongly correlated with faster spreading among countries with reported cases, with more rapid spread in Asia at the early stage of the pandemic, demonstrating that global connectedness and geographic location were significantly associated with the global spread of COVID-19.

While globalization indicator and location are distinct traits irrespective of pandemic activity, countries can improve capabilities to prevent, delay, detect, and respond to public health emergencies, as outlined in International Health Regulation [26]. In the context of the current pandemic, three months after the first reported cases, 38% of the “final” 24 countries reported in-country capacity to test for SARS-CoV-2, whereas most other countries exported or planned to export samples for testing. The limited COVID-19 testing in these countries likely affected their ability to timely detect and report newly imported or subsequent new domestic COVID-19 cases.

In addition to testing, active case-finding and surveillance systems are essential for detecting, monitoring, and curbing transmission of new outbreaks in a country. For COVID-19, WHO primarily recommended using existing ILI/SARI surveillance systems and reporting to GISRS platform [27] and such systems have been adopted by the U.S. and countries in Africa and Europe [28–30]. We examined the existing syndromic surveillance systems for influenza, ILI and SARI based on data reported to WHO Global Influenza Programme in the past 12 months and noted that 7 (29%) of the 24 countries did not report any influenza surveillance data to WHO. The lack of existing influenza surveillance systems in several countries may affect their ability to track new infections after arrival, which would subsequently affect their ability to develop effective and responsive infection prevention and control measures. In addition to influenza surveillances, other syndromic respiratory disease surveillance platforms or methods (e.g. event or community-based surveillance) could also be leveraged for COVID-19 surveillance to test and monitor community spread and detect signals of respiratory symptoms commonly associated with COVID-19. We could not determine the availability of surveillance and testing data from other national sources (e.g., country Ministry of Health).

Border control measures have been commonly used in past pandemics and epidemics to contain and slow the global spread of infectious diseases [11, 31]. For COVID-19, WHO had advised against the implementation of travel restrictions and border closures, because they may be ineffective, divert resources from other interventions, and have a negative impact on social, economic, and assistance activities [32]. The now-well-known asymptomatic and pre-symptomatic transmissions could further decrease the effectiveness of border control measures in preventing the introduction of COVID-19. A review found that entry screening measures had identified very low number of cases for the 2009 H1N1 Influenza Pandemic, 2014/2015 Ebola, and 2002/2003 SARS [33]. Despite the apparent ineffectiveness of border screening measures in identifying active cases, the study also summarized potential important concomitant positive effects, including discouraging ill persons from traveling, raising awareness and educating the traveling passengers, providing contacts of public health authorities to travelers in case they develop symptoms, collecting information for contact tracing, even though these impacts are difficult to evaluate [33].

For the COVID-19 pandemic, several studies have assessed the effectiveness of border measures and found that various border control measures, such as border closure and travel restrictions, had curbed regional or global spread of COVID-19, especially at the early stage of the pandemic [10, 34]. To assess the potential risk of imported cases, we calculated the annual per capita inbound visitor arrivals in the 24 countries using the most recent data reported in 2017 or 2018 [18]. Most of these countries may have a high risk of imported cases due to the volume of foreign visitor to many of the countries, particularly for the Pacific island nations, had they not taken any preemptive border control measures. All 24 countries under study had implemented at least two border control measures against the entry of COVID-19. Some enacted border measures (e.g., travel restrictions and border screenings) as early as January 2020, with more than half of the countries closing their air, land, and sea borders by the end of March. As the global spreading continued, 10 of 24 countries reported their first cases in April (8) and May 2020 (2), 4 reported near the end of 2020, and 10 countries had not reported any cases to WHO as of 18 February 2021. Although this study cannot directly evaluate the effectiveness of the specific measures, proactive implementation of border control measures likely contributed to slowing or preventing the infection across the borders, given the factors discussed above.

There is increasing evidence that asymptomatic and pre-symptomatic transmissions of COVID-19 played a key role in the initiation and acceleration of the outbreaks in other countries [35]. Therefore, while our analysis focused on delaying, detecting, and monitoring the importation of identified cases, it is equally vital for countries to prepare for the potentially undetected arrival of the disease. National strategies and designated task forces can guide and coordinate countries in implementing preparedness activities. The domestic control measures implemented by these 24 countries (e.g., preemptive banning of large gatherings and school or business closures) may have helped to curb the potential spread of undetected infections. Given the reported extreme burdens on healthcare resources and shortage of PPE in countries with widespread outbreaks, it is even more urgent for countries with limited capacity to carry out proactive preparedness activities, such as healthcare worker trainings, acquisition of additional PPE, and designation of local quarantine facilities.

The delay of disease introduction can provide countries a window of opportunity to prepare and implement preparedness strategies. In observing wide-spread transmission of COVID-19 in other countries, the global community accrued a substantial amount of knowledge on disease etiology, detection, treatment, and infection prevention and control measures. Less developed countries with fragile health systems can utilize the knowledge gained and resources shared by the global community through close collaborations and information sharing, to improve outbreak preparedness. To improve early detection, countries can increase in-country testing capacity, testing kits, equipment and supplies, laboratory capacity, and training. For timely and accurate monitoring, countries can improve surveillance capacity by utilizing and adapting existing surveillance systems and participating in international surveillance networks. Additionally, transparent information sharing, and effective communication and outreach, can all aid in the improvement of outbreak preparedness. Moreover, international coordination, as exemplified by the recently formed African Task Force for Coronavirus Preparedness and Response [36, 37], can substantially expand capacities, preparedness and responses on multiple workstreams, including laboratory diagnosis, surveillance, infection prevention and control, clinical treatment, risk communication, and supply chain and stockpile management. Lastly, the global situation continues to evolve despite the availability of COVID-19 vaccines. New challenges continue to emerge, such as the new variants [38, 39]. Therefore, it is essential for the global community to continue to improve and prioritize the capacities needed to prevent, detect, and respond, not only for COVID-19, but also for future global outbreaks.

Our analysis has several limitations. First, the analytic methods are largely observational and qualitative in nature. Therefore, this report cannot determine quantitatively the relative contributions from each factor and is not an evaluation of the effectiveness of such factors in delaying COVID-19 introduction. Nevertheless, the subsequent developments (e.g., significantly delayed introduction or COVID-free status a year after the pandemic declaration) indicated that proactive border measures, global connectiveness, and geographic aspect may be the main contributing factors. Second, data collection via broad web-based search may overlook relevant information, because of the search platforms used, vast amount of information, and/or language translation limitations. Third, we focused on the presence of ILI/SARI surveillance based on WHO’s recommendations and could not assess the availability of other surveillance platforms for the detection and surveillance of COVID-19 cases due to lack of public information. Lastly, potential non-reporting in selected countries can affect the findings and interpretation of data from those countries.

## Conclusions

The limited testing capacity in these countries suggests that many may have lacked the capability to timely detect and monitor coronavirus infections. Geographic location and global connectedness were associated with temporal spreading of COVID-19 globally and may have delayed the importation of cases to these countries. Early implementation of border measures, such as travel restrictions, border closures, and traveler quarantine and screening, may have contributed to delaying the introduction of COVID-19 into these countries, particularly for countries traditionally with a large volume of inbound foreign visitors. The overall low economic status and limited health care resources in these countries demonstrate the importance of early actions to deter the introduction and spread of deadly infectious diseases. The delayed introduction can provide a window of opportunity to improve and implement preparedness strategies, such as increasing disease detection and surveillance capacity. Furthermore, close collaboration with and participation in WHO and other international networks and consortiums as well as transparency in information sharing and exchanges, are essential to enable and improve the preparedness for global outbreaks, particularly for LMICs. Finally, as the global situation continues to evolve, it is essential for countries to continue to improve and prioritize the capacities to rapidly prevent, detect, and respond, not only for COVID-19, but also for future outbreaks.

## Supplementary Information

**Additional file 1:** **Table S1.** COVID-19 Testing Capability and Capacity Summary. **Table S2.** Travel Restrictions and Border Closures Summary. **Table S3.** Screening and Travel Quarantine Summary. **Table S4.** Preparedness Activities Summary.

## Data Availability

All data generated or analyzed during this study are either included in this published article and its supplementary information files or are available from the corresponding author on reasonable request.
