# Supplementary material for: Countries with delayed COVID-19 introduction – characteristics, drivers, gaps, and opportunities
Source: Global Health. 2021 Mar 17;17:28. doi: 10.1186/s12992-021-00678-4 (PMC7968140; doi:10.1186/s12992-021-00678-4)
Supplement: Supplementary file 1 — Additional file 1: Table S1. COVID-19 Testing Capability and Capacity Summary. Table S2. Travel Restrictions and Border Closures Summary. Table S3. Screening and Travel Quarantine Summary. Table S4. Preparedness Activities Summary. [file 12992_2021_678_MOESM1_ESM.docx]

**SUPPLEMENTARY MATERIALS**

**Countries with Delayed COVID-19 Introduction – Characteristics, Drivers, Gaps, and Opportunities**

Zheng Li, PhD^*^, Cynthia Jones, MPH, Girum S. Ejigu, DVM, Nisha George, MPH, Amanda Geller, MPH, Gregory Chang, MPH, Alys Adamski, PhD, Ledor Igboh, MPH, Rebecca D Merrill, PhD, Philip Ricks, PhD, Sara Mirza, PhD, Michael Lynch, MD

*Centers for Disease Control and Prevention (CDC), COVID-19 Response, Atlanta, GA, USA*

* Corresponding Author:

Zheng Li, PhD, MPH

4770 Buford Highway

Atlanta, GA 30341, USA

[ZJLi@cdc.gov](mailto:ZJLi@cdc.gov)

+1) 770-488-7940

Contents

[Table S1. COVID-19 Testing Capability and Capacity Summary 2](#_Toc47208504)

[Table S2. Travel Restrictions and Border Closures Summary 3](#_Toc47208505)

[Table S3. Screening and Travel Quarantine Summary 5](#_Toc47208506)

[Table S4. Preparedness Activities Summary 8](#_Toc47208507)

[REFERENCES 18](#_Toc47208508)

## Table S1. COVID-19 Testing Capability and Capacity Summary

| **WHO Region** | **Country** | **Description** |
| --- | --- | --- |
| AFRO | Botswana(1) | Yes. Botswana National Health Lab could test up to 500 specimens per day. |
|  | Burundi(2, 3) | Yes. The Ministry of Public Health announced that the national lab had the reagents, lab equipment, and skills to test COVID-19 samples. |
|  | Comoros(4, 5) | No, unknown if exporting. WHO offered equipment for disease surveillance, including medical kits with lab equipment to be used for monitoring COVID-19 |
|  | Lesotho(6) | No. Samples from suspected cases were taken to South Africa for testing |
|  | Malawi(7, 8) | Yes - Malawi had 3 testing laboratories and more than 20,000 donated test kits. |
|  | Sao Tome and Principe(9, 10) | No. Exports testing to Senegal and/or Gabon. However, they are awaiting 1000 rapid test kits from South Africa and have contacted WHO about urgently dispatching a promised screening laboratory. |
|  | Sierra Leone(11, 12) | Yes. Sierra Leone has 3 lab facilities capable of testing at least 35 per 1 million people daily. |
|  | South Sudan(13, 14) | Yes. South Sudan had a lab capable of testing for COVID-19 and 20,000 donated test kits. |
| EMRO | Yemen(15) | Yes – as of 29 March 2020, Dr. Saeed Al Shaibani, WHO lab expert, indicated that Yemen’s two central laboratories in Sana’a and Aden are able to perform COVID-19 testing on thousands of samples. |
| EURO | Tajikistan(16) | Yes – as of 9 April 2020, laboratories are fully prepared to detect coronavirus infection and its symptoms. |
|  | Turkmenistan(17) | Yes – The Center for the Prevention of Infectious Diseases and the Center for Public Health and Nutrition are fully equipped with reagents for diagnosing these types of diseases and consumables |
| SEARO | North Korea(18, 19) | Yes - Central Sanitary Quarantine Station, which has established a nationwide sample transportation system, has sufficient material and technical foundation to confirm the presence of suspects |
| WPRO | Cook Islands(20) | No capacity, testing sent to New Zealand |
|  | Kiribati(21) | No capacity, tests sent to Australia |
|  | Marshall Islands(22) | No capacity, tests sent to Guam |
|  | Micronesia(23) | No capacity, tests sent to Guam |
|  | Nauru(24) | No capacity, tests sent to Australia |
|  | Niue | No information given |
|  | Palau(25) | No capacity, tests sent to Guam |
|  | Samoa(26) | In development. As of 9 April 2020, exporting to Australia and New Zealand, but GeneXpert is available and will be functional mid-late April |
|  | Solomon Islands(27) | No capacity, tests sent to Australia |
|  | Tonga | No information given |
|  | Tuvalu | No information given |
|  | Vanuatu(28) | No capacity |

## Table S2. Travel Restrictions and Border Closures Summary

| **WHO Region** | **Country** | **Description of Selected Measures** |
| --- | --- | --- |
| AFRO | Botswana(29-32) | 16 March 2020: All travelers to Botswana from high risk countries are barred entry; visa issuance to travelers from high-risk countries is suspended and visas are cancelled; postponement of government travel to affected countries; residents of Botswana advised to postpone all non-essential local travel  16 March 2020: Parts of the border with South Africa close  24 March 2020: Travel to neighboring countries restricted with the exception of the movement of goods and services  2 April 2020: No person shall enter Botswana other than a citizen or non-citizen resident of Botswana |
|  | Burundi(33, 34) | 5 March 2020 - recommendation for Burundians to limit travel to affected countries  20 March 2020: Suspension of all international flights  4 April 2020: Extension of international flight suspension  No specific statement of border closure |
|  | Comoros(4, 35-37) | 20 March 2020: entry suspended for travelers from all countries that have reported more than 10 cases  18 March 2020: Sea travel between islands suspended  23 March 2020: All international flights suspended  30 March 2020: Border closure |
|  | Lesotho(38, 39) | 19 March 2020: Certain border points are closed  26 March 2020: All unnecessary trips out of the country should be curtailed |
|  | Malawi(40, 41) | 24 March 2020: All non-resident foreign nationals from the following high-risk countries will be denied entry: all countries in the European Union, China, Iran, South Korea, Democratic Republic of Congo, Norway, United Kingdom, Switzerland, Malaysia, Australia, Turkey, Thailand, Israel, United States, South Africa, Canada, Pakistan, Brazil, Saudi Arabia, Ecuador, Chile, and Japan.  1 April 2020: Ban on all commercial international flights  No specific statement about land/water borders |
|  | Sao Tome and Principe(9, 42) | 20 March 2020: all non-resident foreign nationals were banned from entering the country  21 March 2020: São Tomé and Príncipe closed its airspace for 15 days. Charter flights and cruise ships were prohibited from landing or docking at the country’s airports/ports.  8 April 2020: All sea and air connections between the islands of São Tomé and Príncipe were suspended, except for health emergency situations duly authorized by the Prime Minister and Head of Government. |
|  | Sierra Leone(43, 44) | 16 March 2020: All government officials were directed to suspend all foreign travel, while citizens were strongly encouraged to postpone all overseas travel.  22 March 2020: Lungi International airport was closed to all passenger flights.  27 March 2020: Sierra Leone closed its land and sea borders except for essential commodities |
|  | South Sudan(45) | 19 March 2020: South Sudan suspended direct international flights as well as entry via river and dry ports from countries affected by COVID-19. Only South Sudanese citizens and residents holding valid permits would be allowed to enter the country.  23 March 2020: South Sudan closed Juba and all other international airports and canceled all incoming and outgoing international flights as of midnight. The only exceptions to these flights include those used for emergency purposes or humanitarian aid, transporting food, or those flying to foreign destinations through South Sudan air space. Passenger buses traveling into and out of South Sudan also ceased operations as of midnight, and only cargo buses and trucks transporting food and fuel would be allowed to cross land borders. |
| EMRO | Yemen(46, 47) | 29 February 2020: Non-Yemeni nationals with recent travel to China may be refused admission into the country.  28 March 2020: Passenger flights have been suspended and all land entry points have been closed in Yemen. Only cargo imports are allowed into the country following health checks. |
| EURO | Tajikistan(48) | Limited information. As of 9 April 2020, the Dushanbe International Airport was closed. Appears Airline offering repatriation flight for travelers to Frankfurt; there is a waitlist. |
|  | Turkmenistan(17, 49) | 25 February 2020: Control of travelers from affected countries; Limited departures of Turkmen citizens to states with disease foci  3 March 2020: Request from government for foreign travelers to limit travel to Turkmenistan for business or private purposes until 1 April 2020; restricted entry of travelers from COVID-19 affected countries  2 April 2020: All international flights have been cancelled and all land borders have been closed. |
| SEARO | North Korea | No travel restrictions stated  No border closure stated |
| WPRO | Cook Islands(20, 50, 51) | 16 March 2020 – Border closed to anyone besides New Zealand residents  24 March 2020 - Avoid or defer non-essential and non-urgent travel overseas and to the Pa Enua |
|  | Kiribati(52) | No border closure |
|  | Marshall Islands(53) | 2 February 2020 – suspension of all persons travelling via air or sea from affected countries (China, Macao, Hong Kong) for 30 days, which grew to restrict persons from countries with high # of cases.  26 February 2020 - Citizens are strongly advised to not travel abroad  - total suspension of international travelers until 5 May 2020. |
|  | Micronesia(54, 55) | 14 March 2020 - All citizens prohibited from outbound travel to COVID-19 countries, states, and territories. Citizens wishing to travel outbound must acquire the Government’s approval. Cargo ships still allowed to bring in supplies.  19 March 2020 - Non-FSM citizens discouraged to traveling to FSM  Travel to state of Chuuk is banned. Travel to Pohnpei is only allowed for Pohnpei state residents with exceptions for medical capacity building. |
|  | Nauru(56) | 17 March 2020 - flights to and from Nauru will be immediately reduced to once a fortnight to and from Brisbane only. All other passenger flights will be suspended until further notice. Air freight will continue with strict quarantine measures |
|  | Niue(57) | 3 February 2020 -All travelers who have been in or travelled to China within the last 30 days must spend 14 days in a country free from COVID-19 and acquire an official medical clearance.  Cancellation of all government travel to China and other countries affected by COVID-19  1 April 2020 - only Niue residents are allowed into Niue |
|  | Palau(58) | February 2020 - All fights related to China, Hong Kong, and Macau have been suspended until the end of April. Cruise ships from affected countries are banned from docking unless captain submits proof  17 March 2020 - Everyone arriving into Palau must have a health declaration form |
|  | Samoa(59) | 25 March 2020 - Samoa airport COVID-19 lockdown. All flights in and out of Samoa are suspended, all borders closed. |
|  | Solomon Islands(60) | 13 March 2020 - All travelers and crew from affected countries will be subject to risk-based assessments by the Solomon Island Government. May be subject to 14-day monitoring by government officials. No travel restriction on all government officials to COVID-19 restricted countries.  27 March 2020 - No entry of non-citizens into the Solomon Islands. Ban pleasure entertainment cruise ships. On a state of lockdown, which effectively closes borders. Travel within the country is still allowed. |
|  | Tonga(56) | 16 March 2020 - restricted travel to 60 countries including American and Australia  22 March 2020 - entry restrictions requiring international travelers to spend at least 14 days in self-quarantine in a country free of coronavirus prior to entering Tonga. Banned the arrival of cruise ships and yachts into the country until further notice. |
|  | Tuvalu(56) | 25 March 2020 - Restrictions prohibiting entry to Tuvalu for anyone who has been in China within 30 days of arrival in Tuvalu. This restriction includes entry to seafarers from foreign vessels that have been in China or “a high-risk country” (understood to be countries where coronavirus is present) in the last 30 days. |
|  | Vanuatu(61) | 18 March 2020 - authorities announced that travel to and from countries with more than 100 COVID-19 cases is prohibited  21 March 2020 - Vanuatu’s ports of entry are effectively closed. |

## Table S3. Screening and Travel Quarantine Summary

| **WHO Region** | **Country** | **Description of Selected Measures** |
| --- | --- | --- |
| AFRO | Botswana(30, 31, 62) | 28 January 2020: Temperature checks and travel history taken to gauge exposure at airports and border crossings  16 March 2020: Batswana and residents returning from high risk countries subject to mandatory 2-week quarantine |
|  | Burundi(33, 34) | 5 March 2020: All travelers from high risk countries undergo 14-day quarantine  12 March 2020: Quarantine extended to travelers from/through entire EU  23 March 2020: airport screening for elevated temperature and health questionnaire regarding last three weeks of travel |
|  | Comoros(4, 63, 64) | 28 January 2020: Temperature checks at airports  19 February 2020: First three passengers come out of quarantine  16 March 2020: mandatory quarantine for all travelers from areas at risk  30 March 2020: travelers from countries with confirmed cases may be required to quarantine for 14 days in a country free from COVID-19 |
|  | Lesotho(39, 65) | 6 March 2020: US Embassy reports enhanced screening at points of entry and notes that travelers from affected countries may be instructed to self-isolate for 2 weeks  10 March 2020: Self-isolation of travelers from affected areas (due to lack of necessary infrastructure)  19 March 2020: 14-day quarantine of returning citizens |
|  | Malawi(66) | 17 March 2020: Any traveler exhibiting any symptoms of COVID-19 would be evaluated by medical personnel and may be placed in a treatment facility. Any travelers from the European Union, groups of 10 or more from countries with local transmission, or from countries with ≥700 total confirmed cases, ≥100 confirmed cases within 24 hours or with community transmission but not exhibiting any COVID-19 symptoms would be in self-isolation and followed by a health worker for 14 days.  27 March 2020: Malawian government implemented screenings for all incoming passengers at Kamuzu and Chileka International airports. The passengers would have to complete a questionnaire listing out their recent travel history and any symptoms related to COVID-19. Health teams are stationed at arrival sections of both airports and even use a thermal scanner to check body temperature at Kamuzu. Symptomatic Malawian citizens and permanent residents returning from any of the high-risk countries listed above will be placed in an isolation room at the airport for further medical investigation. Asymptomatic travelers from the high-risk countries must be self-quarantined at home and not in hotels or lodges and followed for 14 days. |
|  | Sao Tome and Principe(9, 67) | 15 March 2020: Travelers were required to be tested at starting points and present proof of test upon arrival  21 March 2020: All São Toméan nationals and foreign residents reentering the country are subject to being quarantined at home or at a hotel for 15 days. |
|  | Sierra Leone(43) | 23 March 2020: All passengers arriving in Sierra Leone at any entry point must fill out a passenger locator card capturing demographic information, travel history and symptoms for COVID-19. Screening at entry included a temperature and symptoms check for all passengers and identification of passengers who are required to be quarantined. Any passenger exhibiting fever >37.5°C, persistent cough or difficulty breathing would be immediately taken to an isolation facility. Sierra Leone has quarantine facilities at 3 major entry points: Lungi International Airport, Jendema land border and Gbalamuya land border. All travelers from any country with >50 confirmed cases of COVID-19 were required to be placed in quarantine for a period of 14 days. Travelers from countries <50 confirmed COVID-19 cases will be documented and followed up for 14 days by surveillance officers or contact tracers. |
|  | South Sudan(13, 68, 69) | 13 March 2020: Enhanced screening measures to reduce spread of COVID-19, including using a thermal scanner at Juba International Airport.  19 March 2020: South Sudanese citizens and residents with valid permits returning from COVID-19 affected countries must be self-quarantined for 14 days. |
| EMRO | Yemen(47) | 29 February 2020: Any traveler exhibiting symptoms related to COVID-19 or may have been exposed to or are possibly infected with COVID-19 may be subject to airport screening. Symptomatic travelers arriving in Yemen may be subject to quarantine. |
| EURO | Tajikistan(48) | 25 March 2020: Citizens arriving to Tajikistan have been quarantined. |
|  | Turkmenistan(49) | 17 February 2020: Travelers from affected countries subject to medical screening, including procedures unrelated to COVID-19 (e.g. HIV testing) and may be subject to involuntary quarantine for an unspecified amount of time. |
| SEARO | North Korea(19, 70) | 30 January 2020: Each of the command posts has examination and quarantine measures at border crossing points such as borders, ports, and airfields, and conducts medical surveillance and medical check-ups for foreign visitors and residents to detect and quarantine patients early.  12-13 February 2020: mandatory quarantine of all foreigners in the country for 30 days |
| WPRO | Cook Islands(50, 71) | 23 March 2020: All new arrivals are required to undergo supervised quarantine for 14 days upon arrival. Visitors may return before the 14-day period with written consent from the Ministry of Health. All recent visitors who arrived in the last two weeks will be contacted to assess their COVID-19 health status. |
|  | Kiribati(52) | Travelers “from countries with ongoing local transmission of novel coronavirus” – which includes the United States — must spend at least 14 days in a country free of the virus before traveling to Kiribati, and to provide a medical clearance to confirm that they are virus-free.  Travelers arriving earlier than the required 14-day waiting period may be subject to quarantine and/or deportation. |
|  | Marshall Islands(53) | 24 January 2020: Screening for COVID-19 and measles through health screening form and possible health screening by staff. |
|  | Micronesia(55) | 11 March 2020: All incoming passengers on flights and vessels will be required to have a medical certificate and will be quarantined at the Dekekhik isolation area for 14 days |
|  | Nauru(24, 56) | 13 March 2020: Using body scanners to detect high body temperature. If individual shows symptoms, they will be transferred to a Coronavirus isolation facility for testing and care.  16 March 2020: All persons traveling to Nauru will be required to spend up to 14 days in approved transition accommodation before being allowed back into the community. |
|  | Niue(72) | 3 February 2020: All Niue residents that have travelled from or through China will be self-quarantined for 14 days  24 March 2020: All persons who travelled to or arrived in Niue after 19 March 2020 to be held in self-isolation. If arriving on NZ784 then individual must self-isolate in government approved location |
|  | Palau(73, 74) | 17 March 2020: Any citizen travelling from COVID-19 affected countries must submit comprehensive health screening, self-quarantine for 14 days. |
|  | Samoa(59) | 7 March 2020: All passenger and crew to be screened upon arrival, must have health declaration card (health screening 3 days before entering Samoa), All travelers from COVID-19 affected countries must undergo 14 days self-quarantine at country of last port followed by medical examination |
|  | Solomon Islands(60) | 27 March 2020: All travel is suspended  28 March 2020: All recent travelers are required to be quarantine |
|  | Tonga(56) | 4 February 2020: Travelers from US, China, Australia, Italy and South Korea required to undergo 14-day quarantine.  22 March 2020: Entry restrictions requiring international travelers who either traveled from or through a list of high-risk countries to spend at least 14 days in self-quarantine in a country free of coronavirus prior to entering Tonga.  International flights and vessels entering Tonga are required to use Tongatapu as the first port of entry for border screening before traveling to outer islands. |
|  | Tuvalu(56) | 25 March 2020: Health screening will be conducted at Funafuti airport and seaport and may also be conducted at Nausori (Fiji) Airport and Tarawa (Kiribati) Airport. Travelers who have been in a “high-risk country” must obtain a medical clearance three days prior to entering Tuvalu and must remain in a country other than those listed as “high-risk” for at least five days before reentering Tuvalu. |
|  | Vanuatu(28, 61) | 12 March 2020: Aneityum Island was quarantined due to the visit of a cruise ship that had passengers who later had COVID-19. Recent visitors to Aneityum considered contacts were also quarantined. Quarantine was lifted on 26 March 2020 for Aneityum Island and for contacts. |

## Table S4. Preparedness Activities Summary

|  | **Country** | **Description** |
| --- | --- | --- |
| Preparedness plan or strategy | | |
| AFRO | Botswana(75) | As of 19 March 2020: Committee meetings since 16 March 2020; Preparedness and Response team and High Level National Committees formed; all mitigation strategies + communications strategy |
|  | Burundi | No information available |
|  | Comoros(76) | As of 25 February 2020: With WHO support, the MOH developed a preparedness and response plan for COVID-19 |
|  | Lesotho(39, 77) | As of 19 March 2020: National Command Center for COVID-19 identified  As of 20 March 2020: Newly formed Technical Team at Manthaiseng National Command Centre formulated an integrated plan for awareness, prevention, and management of COVID-19 |
|  | Malawi(78, 79) | 8 April 2020: official launch of national COVID-19 Preparedness and Response Plan that is expected to cost around US $203 million (Malawian kwacha 150 billion), of which only US $9.1 million have been funded thus far.  Funding from UK Aid to be used for airport & border screenings, HCW training, emergency treatment units with medical and intensive care supplies, and WASH facilities |
|  | Sao Tome and Principe(80) | $2.5 million IDA grant is expected to be to put towards the following measures: Support preparedness and response to disease outbreaks and emergencies at both national and subnational levels; Enhance surveillance capacity for early detection, case management and contact tracing; Support health promotion and community mobilization; Adopt a “One Health” approach to improve coordination mechanism for public health emergency activities |
|  | Sierra Leone(11, 81) | On 18 March 2020, the Ministry of Health and Sanitation of Sierra Leone took the following enhanced public health measures to prevent COVID-19: Activate the Emergency Operations Centre to Level 2 to coordinate initial preparedness and response; Streamline standard operating procedures to cover screening, quarantine, laboratory, and data management for persons from places affected by COVID-19; Position expert contact tracers and disease surveillance units in all district levels; Implement handwashing procedures at most medical institutions and encourage private businesses, public institutions and public transportation operators to provide handwashing facilities and/or hand sanitizers.  On 24 March 2020, President Bio declared a state of public emergency for a period of 12 months. |
|  | South Sudan(46, 82) | South Sudan established a High-Level Task Force Committee to address preparedness needs for COVID-19. A COVID-19 Country Preparedness and Response Plan was developed, covering a six-month period to September 2020.  31 March 2020: The Undersecretary of Health indicated that the South Sudanese government has done the following to prepare for the pandemic: Place measures to identify, detect, confirm and respond to potential cases of COVID-19 in the country; Establish an isolation and treatment center and train Rapid Response Teams and frontline health workers to handle COVID-19 cases; Setup screening points at border entry points; Conduct active surveillance at community and health facilities; Participate in community awareness activities with help of partners |
| EMRO | Yemen(46, 83) | UN/WHO: As of 28 March 2020, the UN Country Team established a Crisis Management Team and Preparedness and Response Plan to coordinate efforts against COVID-19, which include setting up isolation units at Sana’a and Aden airports, preparing mass communication campaigns, identifying quarantine facilities, and procuring soft-skin ambulances to transport infectious disease cases. WHO purchased 76 ventilators to be dispatched to health clinics in Yemen and plans to obtain more ventilators.  World Bank: A bulk of the IDA grant ($23.4 million) will be used towards procuring medical supplies and equipment, training and implementation expenses, and rehabilitating and upgrading existing health facilities. These efforts will initially focus on large communities with high risks of local transmission. |
| EURO | Tajikistan | No information available |
|  | Turkmenistan(17) | As of 25 February 2020: Control centers of the Extraordinary Commission for the Control of the Spread of Diseases were created. Work Plan was created to control and analyze prevention measures |
| SEARO | North Korea(19) | As of 1 February 2020: Establishment of an emergency prevention system and a nation-wide project to prevent COVID-19 under the central emergency control command |
| WPRO | Cook Islands(84) | As if 10 March 2020, Cooks Islands released an Emergency Response Plan to COVID-10 |
|  | Kiribati(85) | State of Public Emergency will allow Kiribati to prepare and manage the impact of this Novel Corona Virus 2019 pandemic |
|  | Marshall Islands(22) | Has a plan through the US government |
|  | Micronesia(55) | Creation of Corona Virus task force – review and assess the conditions within the State relating to the spread of COVID-19 and to submit an action plan |
|  | Nauru(24) | Nauru Government Taskforce Council created to prepare for COVID-19. President order issued prohibiting hoarding and price inflation |
|  | Niue(57) | 24 March 2020: Disaster council activated |
|  | Palau(74) | Activation of Public Health Emergency Operations Plan, Certification of unavoidable public health emergency |
|  | Samoa(86) | Per the State of National Emergency, the Sector Preparedness and Response Matrix Consolidated for Corona Virus Pandemic was activated |
|  | Solomon Islands(87) | Proclaimed state of emergency giving the president emergency powers under the Emergency Powers (COVID-19) Regulations 2020 |
|  | Tonga(88) | Proclaimed state of emergency for coronavirus |
|  | Tuvalu(89) | Proclaimed state of emergency for coronavirus |
|  | Vanuatu(90) | Yes, as of 17 March 2020. State of emergency, surveillance grown, etc. |
| Mass gathering restriction | | |
| AFRO | Botswana(30, 31) | 16 March 2020: suspension of gatherings of >100  2 April 2020: gathering of >2 prohibited |
|  | Burundi | No information available |
|  | Comoros(35, 63) | 16 March 2020: Public festivities and gatherings suspended; Wedding ceremonies of >20 people banned  30 March 2020: Suspension of collective prayers; closure of schools and sports centers |
|  | Lesotho(91, 92) | 18 March 2020: Encouragement not to crowd together at festivals or other functions  27 March 2020: gatherings are prohibited except funerals of 50 people or less |
|  | Malawi(93, 94) | 27 March 2020: All government employees are banned from attending regional and international meetings as well as all social, cultural, political and religious events. All government meetings with over 100 people have been banned and all international meetings have been suspended.  9 April 2020: All social, cultural and religious events and sporting activities are suspended. Funerals must comply with social distancing restrictions and can be attended by no more than 50 people. |
|  | Sao Tome and Principe(9, 42) | 26 March 2020: all cultural, recreational or religious public gatherings were not allowed.  8 April 2020: All meetings with more than 10 people were prohibited. |
|  | Sierra Leone(12, 43) | 16 March 2020: All public gatherings with more than 100 people were banned.  23 March 2020: Churches and mosques were closed, and public gatherings were canceled. |
|  | South Sudan(95) | 20 March 2020: President Kiir suspended all planned sporting, religious, socio-cultural and political events for a period of 6 weeks. |
| EMRO | Yemen(96) | As of 2 April 2020, Yemen has not imposed a ban on mass gatherings and continues to keep mosques open for Friday prayers. |
| EURO | Tajikistan | No information available |
|  | Turkmenistan | No information available |
| SEARO | North Korea(19) | 3 March 2020: open air or public gatherings restricted until state of emergency is lifted |
| WPRO | Cook Islands(50) | 23 March 2020: discouraging gatherings of more than 10 people. Residents are also asked to avoid sporting, church and cultural events, and any gatherings at indoor venues where spacing between people of more than two meters cannot be provided. |
|  | Kiribati | None |
|  | Marshall Islands | No information available |
|  | Micronesia | No information available |
|  | Nauru | No, as of 31 March 2020 has not banned mass gatherings |
|  | Niue | No information available |
|  | Palau(74) | 17 March 2020: discouraging of gatherings of 50 people or more |
|  | Samoa(86) | 26 March 2020: State of Emergency Amendment - No gathering of more than 5 people at any public place |
|  | Solomon Islands | No information available |
|  | Tonga(97) | 29 March 2020: national lockdown |
|  | Tuvalu(89) | During state of emergency declared on 20 March 2020, public gatherings are restricted to no more than 10 people |
|  | Vanuatu(90) | 26 March 2020 in press release - Prohibits social gatherings of more than 5 people |
| School closure | | |
| AFRO | Botswana(30, 98) | 18 March 2020: All schools will close from 23 March 2020 until further notice  2 April 2020: All schools closed during the state of public emergency |
|  | Burundi(99) | 25 March 2020: Few school closures, which the government called hasty (Belgian school and French school of Bujumbura) |
|  | Comoros(35) | 30 March 2020: Closure of schools |
|  | Lesotho(39) | 19 March 2020: all schools closed until 20 April 2020 |
|  | Malawi(93) | 27 March 2020: All schools and universities are closed until further notice. |
|  | Sao Tome and Principe(9) | As of 26 March 2020, all public and private schools in the country were closed. |
|  | Sierra Leone(43) | All schools were closed as of 27 March 2020. |
|  | South Sudan(95) | 20 March 2020: South Sudanese government announced the immediate closure of all public and private educational institutions and health science institutes for a period of 30 days. |
| EMRO | Yemen(100) | 22 March 2020: All schools were required to closed and parents were encouraged to keep their children at home. |
| EURO | Tajikistan | No information available |
|  | Turkmenistan | No information available |
| SEARO | North Korea(101) | 6 March 2020: School holidays extended |
| WPRO | Cook Islands(102) | As of 22 March 2020, school holiday will last 2 weeks. As of 29 March 2020, schools closed until 19 April 2020. |
|  | Kiribati(103) | 28 March 2020: State of Public Emergency and partial lockdown declared, and schools are closed until 16 April 2020. |
|  | Marshall Islands(104) | 26 March 2020: public schools go on early spring break and students must stay home and follow strict curfew hours (30 March 2020 – 3 April 2020). |
|  | Micronesia | No information available |
|  | Nauru(24) | No closures of school have been announced as of 24 March 2020. |
|  | Niue(57) | 23 March 2020: School holidays started early and will return when government makes an official announcement. |
|  | Palau(105) | 31 March 2020: Ministry of Education announced that schools will remain closed from 6-17 April 2020 |
|  | Samoa(86) | 26 March 2020: State of Emergency Amendment - All schools are to close until further notice |
|  | Solomon Islands | No information available |
|  | Tonga(106) | 27 March 2020: all schools in Tonga will close for 2 weeks |
|  | Tuvalu | No information available |
|  | Vanuatu(107) | 21 March 2020: All schools located in several named provinces are closed from 23 March 2020 until the order is revoked. |
| Business closure | | |
| AFRO | Botswana(30, 108) | 20 March 2020: Closure of bars, discotheques, nightclubs; restaurant hours are limited |
|  | Burundi | No information available |
|  | Comoros | No information available |
|  | Lesotho(92) | 27 March 2020: Non-essential businesses closed |
|  | Malawi(94) | 9 April 2020: All entertainment and recreational facilities have been mandated to close, and all construction work and mobile markets have been suspended. All eateries are only allowed to provide take away services. Only essential health and security services and agricultural activities are allowed to continue. |
|  | Sao Tome and Principe(42) | 8 April 2020: Closure of all restaurants, bars, cafes, pastries and mobile homes, with the exception of those with home delivery services. |
|  | Sierra Leone(109) | 1 April 2020: All Luma markets have been banned until further notice. President Bio also ordered a 72-hour national lockdown starting on 5 April 2020, requiring all individuals except for those providing essential services (health, security and media personnel) to stay at home. |
|  | South Sudan(95) | 28 March 2020: The government of South Sudan ordered all non-essential businesses to close as of 28 March 2020. |
| EMRO | Yemen | No information available |
| EURO | Tajikistan | No information available |
|  | Turkmenistan | No information available |
| SEARO | North Korea | No information available |
| WPRO | Cook Islands(20, 110) | 23 March 2020: Cancellation of all liquor licenses to prevent congregation at bars/nightclubs  25 March 2020: Restaurants recommended to do take-out only. Main market, Punanganui, partially closed and can only sell produce. |
|  | Kiribati | No information available |
|  | Marshall Islands | No information available |
|  | Micronesia | No information available |
|  | Nauru | No, as of 31 March 2020 has not closed businesses. |
|  | Niue | No information available |
|  | Palau | No information available |
|  | Samoa(86) | As of 26 March 2020: State of Emergency Amendment:  · All churches, bars/night clubs, entertainment gatherings are required to close  · Restaurants are take-out only |
|  | Solomon Islands(87) | As of 27 March 2020: Prime Minister National Emergency - In emergency zones, nightclubs, kava bars, bars, and casinos are closed |
|  | Tonga(97) | 29 March 2020: national lockdown |
|  | Tuvalu | No information available |
|  | Vanuatu(111) | As of 31 March 2020: No closures at the moment, all business mandated to have soap and water and Karas are take out only |
| COVID-19 Funding | | |
| AFRO | Botswana(75, 112) | As of 19 March 2020: The Botswana Government allocated P31 million (Botswanan Pula) towards the COVID-19 outbreak.  As of 27 March 2020, the government reportedly injected P2 billion into relief fund. |
|  | Burundi(113) | As of 31 January 2020: While there has been a mention of funding from partners, no amount has been specified. |
|  | Comoros(76) | As of 25 February 2020, 900 million francs national budget; 126 million francs donation from IOC |
|  | Lesotho(39) | As of 19 March 2020: Proposed budget of M60 million for dissemination of information and increasing human resources |
|  | Malawi(78, 79) | On 27 March 2020, the UK Department of International Development (DFID) had provided about 1.7 billion kwacha (£1.8 million ≈ US $2.2 million) to strengthen Malawi’s capacity to prevent a COVID-19 outbreak in the country. On 8 April 2020, the Malawian MOH indicated that US $9.1 million out of US $203 million available so far for its COVID-19 Preparedness and Response Plan. |
|  | Sao Tome and Principe(80) | As of 2 April 2020: The World Bank approved a $2.5 million International Development Association (IDA) grant to assist COVID-19 preparedness and response efforts. |
|  | Sierra Leone(114) | 2 April 2020: World Bank approved a $7.5 million International Development Association (IDA) grant to Sierra Leone for its COVID-19 response. |
|  | South Sudan(46, 115) | As of 27 March 2020, the U.S. Government has provided $8 million to South Sudan for health-related support and supplies to bolster water and sanitation activities.  As of 28 March 2020: South Sudan is a priority country that is included in both UN Humanitarian Response Plan (HRP) and Regional Refugee Response Plan (RRP). The UN Office of the Coordination of Humanitarian Affairs has currently requested about $2.01 billion as part of its COVID-19 Global Humanitarian Response Plan, which is expected to support the multi-sectoral response to COVID-19 and affected economies of HRP countries. |
| EMRO | Yemen(46, 83) | 28 March 2020: Yemen is a priority country that is included in the UN Humanitarian Response Plan (HRP). The UN Office of the Coordination of Humanitarian Affairs has currently requested about $2.01 billion as part of its COVID-19 Global Humanitarian Response Plan, which is expected to support the multi-sectoral response to COVID-19 and affected economies of HRP countries.  2 April 2020: World Bank approved a $26.9million International Development Association (IDA) grant to Yemen to assist its preparedness efforts to fight COVID-19. |
| EURO | Tajikistan(115) | As of 27 March 2020, the U.S. Government provided approximately $866,000 to help prepare laboratory systems, activate case-finding and event-based surveillance, support technical experts for response and preparedness, bolster risk communication, etc. |
|  | Turkmenistan(115) | As of 27 March 2020, the U.S. Government provided approximately $920,000 to help prepare laboratory systems, activate case-finding and event-based surveillance, support technical experts for response and preparedness, bolster risk communication, etc. |
| SEARO | North Korea | No information available |
| WPRO | Cook Islands(71) | 24 March 2020: funding mentioned being passed by parliament for $4.5 million |
|  | Kiribati | No information available |
|  | Marshall Islands(116) | $858,924 to procure GeneXpert COVID-19 Testing Kits. $8.3 billion funding package; $367,000 of which is allocated to RMI |
|  | Micronesia(55) | The Department of Treasury & Administration in cooperation with the Office of Budget began identifying sources for revenue to prepare for outbreak.  Access to Disaster Assistance and Emergency Fund |
|  | Nauru(24) | Australia to send $100,000 to Nauru to purchase test kits and other associated products use for disease prevention. “Buffer fund” from previous administration will finance extra personnel and services for hospital, police, security, and airline. |
|  | Niue(57) | Working with New Zealand for support.  Working with private sector with Chamber of Commerce for funding. |
|  | Palau(74, 117) | Certification of unavoidable public health emergency.  Ministry of health to access the Hospital Trust Fund.  Addition of $916,808 to fund Authorizing up to 6 million in reserve funding to help maintain government services. |
|  | Samoa(118) | World Bank delivered 5.1m to Samoa COVID-19 preparedness.  United States HHS donates $370,246.50 for COVID-19 related activities. |
|  | Solomon Islands(119) | WHO supports set up of new Triage center at National Referral Hospital.  China donated $300,000 to assist SI to buy testing kits and other lab equipment to help build health care capacity. |
|  | Tonga | No information available |
|  | Tuvalu | No information available |
|  | Vanuatu(120) | Resources and materials have been committed or provided by numerous partners |
| Health worker training | | |
| AFRO | Botswana(62) | As of 28 January 2020: Health care worker refresher training commenced  As of 19 March 2020: 27 lab scientists have been trained on specimen collection and shipment |
|  | Burundi | As of 27 March 2020: Qualified lab personnel at national health lab.  No information available on health worker training |
|  | Comoros(121) | As of 26 February 2020: Training for rapid response teams  As of 28 March 2020: Health professional training for preparedness, preparation, and response to the pandemic |
|  | Lesotho(122) | As of 10 March 2020: 2 doctors, 10 nurses trained |
|  | Malawi(8) | 4 April 2020: Currently conducting training for health care workers and community health workers |
|  | Sao Tome and Principe | No information available |
|  | Sierra Leone(12) | As of 19 March 2020: 3 Sierra Leonean doctors fully trained in Brazzaville, Republic of Congo, to handle such infectious diseases will return to the country as part of its COVID-19 preparedness plan. |
|  | South Sudan(123) | 30 March 2020: WHO South Sudan, UNICEF South Sudan and other partners conducted trainings and simulation exercises in Maridi to build and evaluate the capacity of healthcare workers to detect, investigate and respond to COVID-19 cases. |
| EMRO | Yemen(124) | 22 March 2020: The Yemeni Ministry of Public Health and Population launched training programs for over 50 doctors and nurses on COVID-19 case management, including managing infectious individuals and sorting suspected cases within isolation centers and conducting epidemiological surveillance. |
| EURO | Tajikistan | No information available |
|  | Turkmenistan | No information available |
| SEARO | North Korea | No information available |
| WPRO | Cook Islands(71) | Health professional on standby and frontline training on infection control |
|  | Kiribati | No information available |
|  | Marshall Islands(125) | IPC trainings |
|  | Micronesia | No information available |
|  | Nauru | No information available |
|  | Niue | No information available |
|  | Palau | No information available |
|  | Samoa | No information available |
|  | Solomon Islands | No information available |
|  | Tonga | No information available |
|  | Tuvalu | No information available |
|  | Vanuatu(120) | Trainings have been conducted including for medical staff and hotel staff supporting quarantines. Training of healthcare workers on prevention, case management and use of personal protective equipment (PPE) has been conducted. |
| PPE | | |
| AFRO | Botswana(126) | As of 27 March 2020: Donations from Jack Ma: 100,000 masks, 20,000 testing kits, 1,000 protective suits. |
|  | Burundi(3, 99) | As of 25 March 2020: 15,000 masks and 70,000 gloves; donations from partners. |
|  | Comoros(127) | As of 22 March 2020: PPE reported to be available to doctors and nurses. |
|  | Lesotho(6) | As of 27 March 2020: 20,089 test kits, 741 personal protection equipment (PPE), 100,000 surgical masks and 1,111 face shields arrived on Thursday evening from Jack Ma Foundation; local businesses have donated sanitizers, face masks, gloves, and surface sanitizers. |
|  | Malawi(128, 129) | As of 31 March 2020, Malawi received the following donations from China: 1000 protective face masks, 500 disposable medical protective clothing, 300 infrared thermometers, 480 protective medical goggles, 500 pairs of sterilized surgical gloves, 500 pairs of medical isolation shoes. Additional donations from Jack Ma were received. |
|  | Sao Tome and Principe(130, 131) | 1 April 2020: WHO donated about 30 tons of PPE such as masks, suits, tents, gloves and other equipment  28 March 2020: Jack Ma donated masks, gloves, fans, tests and other laboratory materials |
|  | Sierra Leone | No information available |
|  | South Sudan(14, 95) | Securing medical supplies and personal protective equipment has proven challenging as several countries have put strict export restrictions with immediate effect on the number of products (gloves, goggles, mouth caps, coveralls) that can be purchased. However, on 24 March 2020, South Sudan received 100,000 masks and 1000 protective suits and face shields from the Jack Ma Foundation and the Alibaba Foundation |
| EMRO | Yemen(83, 132) | PPE is very limited in the country, and Yemen is dependent on foreign donations and WHO dispatches. On 23 March 2020, Yemen received PPE items and trauma medications and supplies from Saudi Arabia. |
| EURO | Tajikistan | No information available |
|  | Turkmenistan(17) | As of 25 February 2020: Domestic doctors are provided with PPE. |
| SEARO | North Korea(19, 133) | As of 3-4 March 2020: Korean style PPE is being mass produced, including protective clothing, gloves, booties, and gas masks. |
| WPRO | Cook Islands(20) | As part of code yellow response, assessed PPE stock. |
|  | Kiribati | No information available |
|  | Marshall Islands(125) | Eligible to receive PPE from strategic national stockpile. |
|  | Micronesia | No information available |
|  | Nauru(24) | MOH received COVID-19 supplies from WHO under a Plan of Operation agreement. 200 protective goggles, 7000 examination gloves, 385 face masks, 12,700 surgical masks, 20 respirator masks and 200 surgical gowns. |
|  | Niue | No information available |
|  | Palau | No information available |
|  | Samoa(134) | WHO donated medical masks, gloves and surgical gowns for COVID-19 preparedness. |
|  | Solomon Islands(119) | China also sending 125,000 pairs of protective gloves to Honiara. |
|  | Tonga(135) | While WHO is expected to provide PPE, no indications whether this equipment has been received. |
|  | Tuvalu | No information available |
|  | Vanuatu(120) | As of 20 March 2020, plans for PPE stocks and request for financing and procurement of equipment.  As of 1 April 2020, PPE stocks have been received from partners and are being continually reviewed for re-stock. |
| Quarantine/Isolation facility | | |
| AFRO | Botswana | No information available |
|  | Burundi(33, 136) | As of 1 February 2020: all suspected cases taken to Prince Regent Charles Hospital, isolated for 2 weeks  As of 30 March 2020: Quarantined individuals taken to Government Hotel Facility in Bujumbura |
|  | Comoros(137) | As of 25 March 2020: Al-Amal hotel serves as quarantine site |
|  | Lesotho(39) | As of 19 March 2020: Fedix, Likileng in Butha-Buthe as a quarantine facility |
|  | Malawi | No information available |
|  | Sao Tome and Principe | No information available |
|  | Sierra Leone(12) | As of 19 March 2020, there were over 200 people currently under quarantine at various isolation facilities around the country. There was a surge in the number of quarantine cases following the recent traveler quarantine measures. |
|  | South Sudan(95, 138) | As of 29 March 2020: South Sudan imposed a mandatory 14-day quarantine on all travelers returning from countries affected by COVID-19 on 19 March 2020. While there are some isolation/ quarantine areas in cities such as Nimule, the government has found it difficult to enforce quarantine as indicated by reports of 500 South Sudan nationals breaking out of quarantine in a secondary school after arriving from Sudan or 2 South Sudanese individuals from Uganda leaving quarantine and attempting to re-enter the country.  31 March 2020: United Nations High Commissioner for Refugees (UNHCR) is participating in the National COVID-19 Task Force to identify and set up isolation/quarantine wards in refugee camps. |
| EMRO | Yemen | No information available |
| EURO | Tajikistan(139) | 4 February 2020: 2 hospitals have established quarantine centers for people arriving from China.  6 April 2020: 6 medical facilities in Khujand and 12 health facilities in other cities and districts of the region are ready for quarantine. |
|  | Turkmenistan(49) | As of 17 March 2020: Travelers from COVID-19 affected countries, even if asymptomatic, may be taken to an infectious disease hospital or other quarantine facility and be required to spend up to 24 days in the hospital before being permitted to continue their visit. |
| SEARO | North Korea(19) | As of 10 March 2020: quarantine facilities mentioned in news article. |
| WPRO | Cook Islands | No information available |
|  | Kiribati | No information available |
|  | Marshall Islands(53) | Government announced on 15 February 2020. That new eight-bed isolation unit to be built and ready within the next 30 days |
|  | Micronesia(140) | Government designated area, Dekektik isolation area |
|  | Nauru(24) | Isolation facilities include:  Budapest hotel, Menen hotel, and Anibare village  Parts of Menen hotel will be used for individuals showing symptoms  Acute block in RoN Hospital will be used as the treatment station for those requiring hospitalization |
|  | Niue | No information available |
|  | Palau | No information available |
|  | Samoa | No information available |
|  | Solomon Islands(87) | 27 March 2020: Prime Minister National Emergency declared that a designated quarantine station to be made available. |
|  | Tonga | In development |
|  | Tuvalu | No information available |
|  | Vanuatu(120) | TB ward has been renovated to be used for isolation. |

## REFERENCES

1. Botswana Government. @BWGovernment. Together we can beat... 5:30 AM EST, 17 March 2020. Available from: <https://twitter.com/BWGovernment/status/1239846522679963648>.

2. Tuesday, March 24, 2020 Press Release From The Ministry Of Public Health And AIDS Control On COVID-19 Prevention Measures [press release]. Ministere de la Sante Publique et de la Lutte Contre la Sida Burundi, 25 March 2020. Available from: <http://minisante.bi/?p=585>.

3. Policy Responses to COVID-19 [Online]. International Monetary Fund; 2020 [updated 3 April 2020; cited 2020 5 April]. Available from: <https://www.imf.org/en/Topics/imf-and-covid19/Policy-Responses-to-COVID-19>.

4. COVID-19 Information - Madagascar and Comoros [Web page]. U.S. Embassy in Madagascar and Comoros; 2020 [cited 2020 30 March]. Available from: <https://mg.usembassy.gov/u-s-citizen-services/security-and-travel-information/covid-19-information/>.

5. Union of the Comoros Ministry of Health. Signature of the 2020-2021 budget program. 9:08 AM, 20 February 2020. Available from: <https://www.facebook.com/permalink.php?story_fbid=863454010778282&id=320950001695355>.

6. State Of Readiness Of Lesotho On Corona Virus (COVID 19) [press release]. Government of Lesotho, 27 March 2020. Available from: <https://www.gov.ls/wp-content/uploads/2020/03/NATIONAL-COMMAND-CENTRE-REPORT-BACK-COVID-19.pdf>.

7. Public Health Institute of Malawi (PHIM) Epidemiology. Public Health Institute of Malawi - Some of the activities being conducted. Malawi Ministry of Health Facebook page: Malawi Ministry of Health; 2020 30 March 2020. Available from: <https://scontent-den4-1.xx.fbcdn.net/v/t1.0-9/91656624_2746137202108444_7176933150965104640_n.jpg?_nc_cat=109&_nc_sid=110474&_nc_ohc=FyxfARRI_-sAX-BWAlV&_nc_ht=scontent-den4-1.xx&oh=c23b3dfea30f106b94ef99a578b4848b&oe=5EA5F170>.

8. Ministry of Health - Malawi. @malawimoh. Statement by the honorable minister of health Hon Jappie Mhango to the media. 7:08 AM, 4 April 2020. Available from: <https://www.facebook.com/malawimoh/posts/2756339911088173?__tn__=K-R>.

9. COVID-19 Information - Gabon and São Tomé and Príncipe [Web page]. U.S. Embassy in Gabon; 2020 [updated 28 March 2020; cited 2020 30 March]. Available from: <https://ga.usembassy.gov/u-s-citizen-services/coronavirus-update/>.

10. Government of Sao Tome and Principe. @governostp. COVID 19 in São Tomé and Príncipe. 9:19 AM, 6 April 2020. Available from: <https://www.facebook.com/527904611056037/posts/813518779161284/?d=n>.

11. Broadcast by His Excellency, Dr. Julius Maada Bio, President of the Republic of Sierra Leone, on Enhanced Public Health and Safety Measures to Prevent the Corona Virus (COVID-19). [press release]. Freetown, Sierra Leone: Sierra Leone Statehouse, 18 March 2020. Available from: <https://statehouse.gov.sl/wp-content/uploads/2020/03/Statement-by-His-Excellency-Dr-Julius-Maada-Bio-President-of-the-Republic-of-Sierra-Leone-on-Enhanced-Public-Health-and-Safety-Measures-to-Prevent-Coronavirus.-Freetown-Sierra-Leone-18-March-2020.pdf>.

12. Sierra Leone Ministry of Health and Sanitation. Health Minister Outlines Sierra Leone's Corona Virus Readiness. 11:53, 19 March 2020. Available from: <https://www.facebook.com/Ministry-of-Health-and-Sanitation-Sierra-Leone-697016430737560/>.

13. Daily Press Briefing by the Office of the Spokesperson for the Secretary-General [Web page]. United Nations; 2020 [updated 26 March 2020; cited 2020 6 April]. Available from: <https://www.un.org/press/en/2020/db200326.doc.htm>.

14. Jack Ma and Alibaba Foundations' anti COVID-19 medical supplies arrive in South Sudan: Ministry of Foreign Affairs of the People's Republic of China; 2020 [updated 26 March 2020; cited 2020 3 April]. Available from: <https://www.fmprc.gov.cn/mfa_eng/wjb_663304/zwjg_665342/zwbd_665378/t1761256.shtml>.

15. WHO Yemen. @WHOYemen. Meeting with Dr. Saeed Al Shaibani, WHO laboratory expert, speaking on the operational capacity of laboratories in #Yemen as part of the response plan for the new corona virus (KO-19). 6:06 AM, 29 March 2020. Available from: <https://www.facebook.com/watch/?v=571159663749899>.

16. Press Statement Of The Ministry Of Health And Social Protection Of The Population Of Tajikistan [press release]. Ministry of Health and Social Protection of the Population of the Republic of Tajikistan, 6 April 2020. Available from: <https://moh.tj/press-statement-of-the-ministry-of-health-and-social-protection-of-the-population-of-tajikistan/?lang=en>.

17. President of Turkmenistan holds meeting on preventing the penetration of viruses into Turkmenistan: State News Agency of Turkmenistan; 2020 [updated 25 February 2020; cited 2020 25 March]. Available from: <http://tdh.gov.tm/news/articles.aspx&article21616&cat11>.

18. Active Steps Taken in DPRK to Prevent Novel Coronavirus Infection [Web page]. The Rodong Sinmun; 2020 [updated 4 February 2020; cited 2020 7 April]. Available from: <http://rodong.rep.kp/en/index.php?strPageID=SF01_02_01&newsID=2020-02-04-0012>.

19. Korean Central News Agency. Domestic News [Web page]. Chosun Central Communications; 2020 [updated 7 April 2020; cited 2020 7 April]. Available from: <http://www.kcna.kp/kcna.user.article.retrieveNewsViewInfoList.kcmsf;jsessionid=182B963C175FC302C43E2E5CBFC54C95>.

20. Cook Islands moves to Code Yellow Alert [press release]. Cook Islands: Cook Islands Ministry of Health, 24 March 2020. Available from: <http://cit-covid19.s3-ap-southeast-2.amazonaws.com/Media+release_+Cook+Islands+moves+to+Code+Yellow+FINAL.PDF>.

21. Office of Te Beretitenti. @ob.gov.ki. Press Release: Negative COVID-19 results. 6:00 AM, 24 March 2020. Available from: <https://www.facebook.com/pg/ob.gov.ki/posts/>.

22. Rachael Doherty. Fact Sheet: U.S. Assistance to the Republic of the Marshall Islands on COVID-19. Office of the President, Republic of the Marshall Islands; 2020. Available from: <https://www.facebook.com/PresidentOfficeRMI/>.

23. My Fellow Micronesians: Through Peace, Friendship, Cooperation, & Love in Our Common Humanity We Will Emerge From the COVID-19 Pandemic as a Stronger Nation & A Stronger World [press release]. FSM Information Services, 17 March 2020. Available from: <https://www.fsmgov.org/fsmun/pubhealtk.pdf>.

24. Calls for concerted effort to safeguard Nauru form coronavirus. Government Information Office; 2020 24 March. Contract No.: 4-2020/208. Available from: <http://www.naurugov.nr/media/121885/nauru_bulletin__04_24mar2020__208_.pdf>.

25. Potential COVID-19 Case - Public Advised to Stay Calm and Practice Preventive Measures [Web page]. Republic of Palau Ministry of Health; 2020 [updated 31 March; cited 2020 1 April]. Available from: <https://www.palaugov.pw/potential-covid-19-case-public-advised-to-stay-calm-and-practice-preventive-measures/>.

26. Ministry of Health Coronavirus (COVID-19) Update 18/03/2020 [press release]. Samoa Ministry of Health, 18 March 2020. Available from: <http://www.samoagovt.ws/2020/03/ministry-of-health-coronavirus-covid-19-update-18-03-2020/>.

27. Solomon Islands Again Receives another NEGATIVE COVID-19 test Result [press release]. Solomon Islands Ministry of Health and Medical Services, 28 March 2020. Available from: <https://solomons.gov.sb/wp-content/uploads/2020/03/Another-Negative-COVID-19-Test-Result-for-Solomon-Islands.pdf>.

28. Coronavirus disease 2019 (COVID-19) Vanuatu Situation Report 2. Vanuatu: Health Promotions Vanuatu; 2020 22 March. Available from: <https://www.facebook.com/pg/Health-Promotions-Vanuatu-1674266679566197/posts/?ref=page_internal>.

29. Closure of Points of Entry (Borders) Due To the COVID-19 (Corona Virus) [press release]. Botswana Unified Revenue Service, 16 March 2020. Available from: <https://www.facebook.com/OFFICIAL.MOHW.BW/posts/1566822526798137>.

30. Emergency Powers (COVID-19) Regulations, 2020. Government Gazette Extraordinary. 2020 2 April 2020. Available from: <https://www.facebook.com/BotswanaGovernment/posts/2874239925991980>.

31. Public Notice [press release]. Office of the President, Republic of Botswana, 16 March 2020. Available from: <https://twitter.com/BWGovernment/status/1239583690457526272>.

32. Botswana Government. @BWGovernment. To minimize risk of... 12:12 PM, 16 March 2020. Available from: <https://twitter.com/BWGovernment/status/1239585452711186432>.

33. COVID-19 Information - Burundi [Web page]. U.S. Embassy in Burundi; 2020 [updated 4 April 2020; cited 2020 5 April]. Available from: <https://bi.usembassy.gov/covid-19-information/>.

34. Thursday, March 5, 2020: Press Release From The Ministry Of Public Health And Aids Control On COVID-19 Prevention Measures [press release]. Ministry of Public Health and the Fight Against AIDS Burundi, 5 March 2020. Available from: <http://minisante.bi/?p=549>.

35. Address to Nation II of His Excellency AZALI Assoumani President of the Union of the Comoros About COVID 19 [Web page]. Union of the Comoros: Presidency of the Republic - Comoros; 2020 [updated 16 March 2020; cited 2020 24 March]. Available from: <https://beit-salam.km/actualit%C3%A9s/adresse-%C3%A0-la-nation-ii-de-son-excellence-monsieur-azali-assoumani-pr%C3%A9sident-de-l%E2%80%99union-des-comores-sur-le-covid-19.html>.

36. Joint Statement of the Ministries [press release]. Ministry of Health Comoros, 19 March 2020. Available from: <https://www.facebook.com/permalink.php?story_fbid=883136822143334&id=320950001695355>.

37. March 18, 2020 - Further restrictions announced by President Azali to combat the spread of COVID-19.: U.S. Embassy in Madagascar & Comoros; 2020 [updated 18 March 2020; cited 2020 25 March]. Available from: <https://mg.usembassy.gov/u-s-citizen-services/security-and-travel-information/march-16-2020-restrictions-announced-by-president-azali-to-combat-covid-19-2/>.

38. Alert: Lockdown Order By Government of Lesotho [Web page]. US Embassy in Lesotho; 2020 [updated 26 March 2020; cited 2020 30 March]. Available from: <https://ls.usembassy.gov/alert-lockdown-order-by-government-of-lesotho/>.

39. Lesotho Government Identifies National Command Centre For COVID-19 [Web page]. Government of Lesotho; 2020 [updated 19 March 2020; cited 2020 25 March]. Available from: <https://www.gov.ls/lesotho-government-identifies-national-command-centre-for-covid-19/>.

40. COVID-19: Self-Isolation/Quarantine of Travelers [press release]. Government of Malawi Ministry of Health and Population, 24 March 2020. Available from: <https://scontent-den4-1.xx.fbcdn.net/v/t1.0-9/90946440_2737341896321308_4620318017929084928_n.jpg?_nc_cat=105&_nc_sid=110474&_nc_ohc=Pg0eOHwv-xAAX83zecI&_nc_ht=scontent-den4-1.xx&oh=ae7f1ccaa88dd947982f029c2acbc6c8&oe=5EA763F5>.

41. Health Alert - U.S. Embassy Lilongwe, Malawi: U.S. Embassy Lilongwe, Malawi; 2020 [updated 1 April 2020; cited 2020 9 April]. Available from: <https://mw.usembassy.gov/health-alert-u-s-embassy-lilongwe-malawi-3-2-2-2/>.

42. Ministério da Saúde - São Tomé e Príncipe. @governostp. Council of Ministers Announcement. 2:19 PM, 6 April 2020. Available from: <https://www.facebook.com/governostp/posts/813710879142074?__tn__=K-R>.

43. COVID-19 Information - Sierra Leone [Web page]. U.S. Embassy in Sierra Leone; 2020 [updated 23 March 2020; cited 2020 30 March]. Available from: <https://sl.usembassy.gov/covid-19-information/>.

44. Sierra Leone’s President Julius Maada Bio Announces Closure of Land Borders [Web page]. Sierra Leone State House Government webpage: Sierra Leone State House Government Communications Unit; 2020 [updated 27 March 2020; cited 2020 30 March]. Available from: <https://statehouse.gov.sl/sierra-leones-president-julius-maada-bio-announces-closure-of-land-borders/>.

45. Government of South Sudan Ministry Of Health. @MohGoSS.ss. New measures today in preventing and combating coronavirus in South Sudan... 5:35 PM, 23 March 2020. Available from: <https://www.facebook.com/watch/?v=515576652394172>.

46. United Nations Office for the Coordination of Humanitarian Affairs (OCHA). Global Humanitarian Response Plan COVID-19. 2020 28 March. Available from: <https://www.unocha.org/sites/unocha/files/Global-Humanitarian-Response-Plan-COVID-19.pdf>.

47. Health Alert – U.S. Embassy Yemen [Web page]. U.S. Embassy in Yemen; 2020 [updated 29 February 2020; cited 2020 29 March]. Available from: <https://ye.usembassy.gov/health-alert-022920/>.

48. COVID-19 Information - Tajikistan [Web page]. U.S. Embassy in Tajikistan; 2020 [cited 2020 29 March]. Available from: <https://tj.usembassy.gov/covid-19-information/>.

49. COVID-19 Information - Turkmenistan [Web page]. U.S. Embassy in Turkmenistan; 2020 [updated 17 March 2020; cited 2020 25 March]. Available from: <https://tm.usembassy.gov/covid-19-information/>.

50. Additional Measures -Bulletin 1 [press release]. Cook Islands: Ministry of Health Cook Islands, 23 March 2020. Available from: <https://cit-covid19.s3-ap-southeast-2.amazonaws.com/Additional+Measures+Bulletin+1+-23+March+2020.pdf>.

51. Notice to refuse entry into the Cook Islands, (2020). Available from: <https://cit-covid19.s3-ap-southeast-2.amazonaws.com/section+9A+notice+-+16+March+20+final.pdf>.

52. COVID-19 Information - Fiji, Kiribati, Nauru, Tonga, and Tuvalu [Web page]. U.S. Embassy in Fiji, Kiribati, Nauru, Tonga, and Tuvalu; 2020 [cited 2020 1 April]. Available from: <https://fj.usembassy.gov/u-s-citizen-services/covid-19-information/>.

53. COVID-19 Information - Republic of the Marshall Islands: U.S. Embassy of the Republic of the Marshall Islands; 2020 [updated 19 March 2020; cited 2020 1 April]. Available from: <https://mh.usembassy.gov/covid-19-information/>.

54. FSM State of Yap Joins Chuuk & Pohnpei in Prohibiting Aircraft Disembarkation; 14-Day Delay for Cargo Shipping Rescinded in Favor of Strengthened Health & Port Security Protocols [press release]. FSM Information Services: Government of the Federated States of Micronesia, 25 March 2020. Available from: <https://www.fsmgov.org/fsmun/pubhealtg.pdf>.

55. National Declaration Placing the Entire Federated States of Miconiesia under a State of National Public Health Emergency in Connection with the COVID-19 Pandemic [press release]. The President, Federated States of Micronesia, 14 March 2020. Available from: <https://gov.fm/files/Declaration_as_of_March_14.pdf>.

56. Kiribati US Embassy in Fiji, Nauru, Tonga, and Tuvalu. US Embassy Pacific Islands 2020 [Available from: <https://fj.usembassy.gov/u-s-citizen-services/covid-19-information/>.

57. Premier's Statement - Code Yellow [press release]. Office of the Secretary of Government NIUE, 23 March 2020. Available from: <https://www.facebook.com/niue.secgov/posts/106438427668644>.

58. COVID-19 Information - Palau [Web page]. U.S. Embassy in the Republic of Palau; 2020 [updated 24 March 2020; cited 2020 28 March]. Available from: <https://pw.usembassy.gov/covid-19-information/>.

59. Samoa Airport Authority COVID-19 Lockdown: Samoa Ministry of Health; 2020 [updated 25 March 2020; cited 2020 31 March]. Available from: <http://www.samoagovt.ws/2020/03/samoa-airport-authority-covid-19-lockdown/>.

60. Ref: Joint travel advisory No 4 [press release]. Solomon Islands Government, 21 March 2020. Available from: <https://solomons.gov.sb/wp-content/uploads/2020/03/Travel-Advisory-4.pdf>.

61. COVID-19 Information - Papua New Guinea, Solomon Islands, and Vanuatu [Web page]. U.S. Embassy in Papua New Guinea, Solomon Islands, and Vanuatu; 2020 [updated 23 March 2020; cited 2020 25 March]. Available from: <https://pg.usembassy.gov/covid-19-information/>.

62. The Argus Online. @argusonlineBW. Corona virus update. 7:16 AM, 28 January 2020. Available from: <https://www.facebook.com/argusonlineBW/videos/473381273344209/UzpfSTQ1NjY5NzE3NzgxMDY4MzoxNTI1MjgzODc0Mjg1MzM2/>.

63. March 16, 2020 - Restrictions announced by President Azali to combat COVID-19: U.S. Embassy in Madagascar & Comoros; 2020 [updated 16 March 2020; cited 2020 25 March]. Available from: <https://mg.usembassy.gov/u-s-citizen-services/security-and-travel-information/march-16-2020-restrictions-announced-by-president-azali-to-combat-covid-19/>.

64. Union des Comores Ministère de la Santé. Passengers returning to the Comoros... 5:05 AM, 28 January. Available from: <https://www.facebook.com/permalink.php?story_fbid=847784045678612&id=320950001695355>.

65. Health Alert - U.S. Embassy Lesotho: U.S. Embassy in Lesotho; 2020 [updated 6 March 2020; cited 2020 30 March]. Available from: <https://ls.usembassy.gov/lesothos-new-directive-on-coronavirus-international-arrivals-now-asked-to-self-isolate-for-14-days/>.

66. COVID-19 Information - Malawi [Web page]. U.S. Embassy in Malawi; 2020 [updated 27 March 2020; cited 2020 30 March]. Available from: <https://mw.usembassy.gov/u-s-citizen-services/covid-19-information/>.

67. Ministério da Saúde - São Tomé e Príncipe. @MSaudeSTeP. Covid-19: São Tomé without infections but population must be prepared for the worst - government. 17:08, 15 March 2020. Available from: <https://www.facebook.com/MSaudeSTeP/posts/2768527333267349>.

68. COVID-19 Information - South Sudan: U.S. Embassy in South Sudan; 2020 [updated 24 February 2020; cited 2020 30 March]. Available from: <https://ss.usembassy.gov/covid-19-information/>.

69. Government of South Sudan Ministry Of Health. @MohGoSS.ss. For your info... 2:33 PM, 20 March 2020. Available from: <https://www.facebook.com/100129098086981/posts/199079284858628/?d=n>.

70. Brisk Hygienic Information Service and Anti-epidemic Work [Web page]. The Rodong Sinmun; 2020 [updated 13 February 2020; cited 2020 7 April]. Available from: <http://rodong.rep.kp/en/index.php?strPageID=SF01_02_01&newsID=2020-02-13-0001>.

71. Covid-19 - Cook Islands Government elevate border entry requirements [press release]. Cook Islands: Ministry of Foreign Affairs and Immigration - Cook Islands, 24 March 2020. Available from: <https://cit-covid19.s3-ap-southeast-2.amazonaws.com/Covid-19+-+Cook+Islands+Government+elevate+border+entry+requirements.pdf>.

72. Communication update for COVID-19 Niue National Disaster Management Office [press release]. Facebook: Niue Health Department, 24 March 2020. Available from: <https://www.facebook.com/photo?fbid=107836340862186&set=a.102231514756002>.

73. Executive Order No. 436 [press release]. Office of the President, Republic of Palau, 13 February 2020. Available from: <https://www.facebook.com/PalauPresident/posts/1545620792256681>.

74. Certification of Unavoidable Public Health Emergency, (17 March 2020, 2020). Available from: <http://www.palauhealth.org/2019nCoV/MOH_PH_Emergency%20Declaration-03172020.pdf>.

75. Botswana Government. @BotswanaGovernment. Remarks By President Masisi On The Occasion Of A Press Conference Regarding Botswana Preparedness About COVID-19 (19/3/2020). 8:08 AM, 19 March 2020. Available from: <https://www.facebook.com/BotswanaGovernment/posts/2832225860193387>.

76. Union of the Comoros Ministry of Health. The IOC releases 126 Million... 1:43 AM, 25 February 2020. Available from: <https://www.facebook.com/permalink.php?story_fbid=866668407123509&id=320950001695355>.

77. Stakeholders Join Efforts In Mitigation Of COVID-19 [press release]. Government of Lesotho, 20 March 2020. Available from: <https://www.gov.ls/stakeholders-join-efforts-in-mitigation-of-covid-19/>.

78. Ministry of Health - Malawi. @malawimoh. Speech by the Chairperson... 9:52 AM, 8 April 2020. Available from: <https://www.facebook.com/malawimoh/posts/2765225403532957?__tn__=K-R>.

79. UK Aid provides 1.7 billion kwacha for COVID-19 (coronavirus) prevention and preparedness in Malawi [Web page]. UNICEF; 2020 [updated 27 March 2020; cited 2020 8 April]. Available from: <https://www.unicef.org/malawi/press-releases/uk-aid-provides-17-billion-kwacha-covid-19-coronavirus-prevention-and-preparedness>.

80. Sao Tome and Principe to Boost Preparedness for COVID-19 [Online]. The World Bank; 2020 [updated 2 April 2020; cited 2020 8 April]. Available from: <https://www.unicef.org/malawi/press-releases/uk-aid-provides-17-billion-kwacha-covid-19-coronavirus-prevention-and-preparedness>.

81. Sierra Leone’s President Julius Maada Bio declares a State of Public Emergency, says there is no lockdown: State House Media and Communications Unit; 2020 [updated 24 March 2020; cited 2020 7 April]. Available from: <https://statehouse.gov.sl/sierra-leones-president-julius-maada-bio-declares-a-state-of-public-emergency-says-there-is-no-lockdown/>.

82. Media D. Juba, Coronavirus Situational Update. 31 March 2020. Undersecretary, of Health Dr. Makur Matur Speaks about latest on coronavirus on March 31,2020. Available from: <https://www.youtube.com/watch?v=ahVn0hlGVnI>.

83. New US$26.9 Million Grant for Yemen to Fund Emergency Response Activities Related to Coronavirus Outbreak [press release]. The World Bank, 2 April 2020. Available from: <https://www.worldbank.org/en/news/press-release/2020/04/02/new-us269-million-grant-for-yemen-to-fund-emergency-response-activities-related-to-coronavirus-covid-19-outbreak>.

84. Cook Islands emergency response plan to COVID-19. Cook Islands Government; 2020 10 March. Available from: <https://cit-covid19.s3-ap-southeast-2.amazonaws.com/CookIslandsEmergencyResponsePlanCOVID19_Mar2020.pdf>.

85. Office of Te Beretitenti Republic of Kiribati. Kiribati Declares State of Public Emergency and Partial Lock Down in Response to Corona Virus Pandemic. 3/28/2020. Available from: <https://www.facebook.com/ob.gov.ki/posts/1103726013322941>.

86. Funefe'ai Dikaiosune Atoa Tamaalii. Amended State of Emergency Orders for Coronavirus (COVID-19) 26th March 2020 [Web page]. Samoa Ministry of Health; 2020 [updated 26 March 2020; cited 2020 28 March]. Available from: <http://www.samoagovt.ws/2020/03/amended-state-of-emergency-orders-for-coronavirus-covid-19-26th-march-2020/>.

87. Hon. Prime Minister Statement To The Nation On State Of Public Emergency [press release]. Solomon Islands Government, 27 March 2020. Available from: <https://solomons.gov.sb/wp-content/uploads/2020/03/PMs-STATEMENT-TO-THE-NATION-27-3-20.pdf>.

88. Declaration of a public heatlh emergency: Government of the Kingdom of Tonga; 2020 [updated 16 March 2020; cited 2020 20 April]. Available from: <http://www.gov.to/press-release/declaration-of-a-public-health-emergency/>.

89. Proclamation of State of Emergency for the Management and Minimisation of the Impact of Coronavirus (COVID-19), Stat. Under section 35 of the constitution of Tuvalu (20 March 2020, 2020). Available from: <https://twitter.com/TuvaluGov/status/1240940455719604225>.

90. Press Release from the Office of the Prime Minister on the State of Emergency declared by the President of Vanuatu [press release]. Vanuatu: Office of the Prime Minister, 26 March 2020. Available from: <https://covid19.gov.vu/images/Press-Release/Press_statement_from_PMO_on_SOE.pdf>.

91. COVID-19 Declared State of Emergency [press release]. Government of Lesotho, 18 March 2020. Available from: <https://www.gov.ls/wp-content/uploads/2020/03/COVID-19-DECLARED-STATE-OF-EMERGENCY.pdf>.

92. Declaration of COVID-19 State of Emergency Notice. Lesotho Government Gazette. 2020 27 March 2020. Available from: <https://www.gov.ls/wp-content/uploads/2020/03/Lockdown-Gazette-Lesotho.pdf>.

93. Health Alert - U.S. Embassy Lilongwe, Malawi [Web page]. U.S. Embassy in Malawi; 2020 [updated 27 March 2020; cited 2020 30 March]. Available from: <https://mw.usembassy.gov/health-alert-u-s-embassy-lilongwe-malawi/>.

94. Ministry of Health - Malawi. @malawimoh. Press Briefing on COVID-19 by the Minister of Local Government and Rural Development. 12:17 PM, 9 April 2020. Available from: <https://www.facebook.com/malawimoh/posts/2767548476633983>.

95. South Sudan COVID-19 Update. ReliefWeb: United Nations High Commissioner for Refugees; 2020 31 March 2020. Available from: <https://reliefweb.int/sites/reliefweb.int/files/resources/75027.pdf>.

96. The National Center for Health and Population Education and Media - Yemen. @health.ed.yemen1. Happy Friday. 6:30 PM, 2 April 2020. Available from: <https://www.facebook.com/585383058147888/posts/3079612562058246/?d=n>.

97. National Lockdown Notice (29 March 2020, 2020). Available from: <https://twitter.com/TongaPolice/status/1243051632843042817/photo/1>.

98. Press release [press release]. Republic of Botswana Government, 18 March 2020. Available from: <https://twitter.com/BWGovernment/status/1240318319414185984>.

99. Communique from the Government of the Republic of Burundi in the Framework of the Fight Against Corona Virus. [press release]. Presidency of the Republic of Burundi, 26 March 2020. Available from: <https://presidence.gov.bi/2020/03/26/le-communique-du-gouvernement-dans-le-cadre-de-la-lutte-contre-le-coronavirus/>.

100. The National Center for Health and Population Education and Media - Yemen. @health.ed.yemen1. The High Committee to combat diseases has taken a number of decisions, including the closure of schools. 2:28 PM, 22 March 2020. Available from: <https://www.facebook.com/health.ed.yemen1/posts/3054094951276674>.

101. The Rodong Sinmun. Great Efforts Constantly Focused on Hygienic and Anti-epidemic Work: The Rodong Sinmun; 2020 [updated 6 March 2020; cited 2020 7 April]. Available from: <http://rodong.rep.kp/en/index.php?strPageID=SF01_02_01&newsID=2020-03-06-0006>.

102. COVID-19 Cook Islands Response Ministry of Education Media Release [press release]. Cook Islands Ministry of Education, 22 March 2020. Available from: <https://cit-covid19.s3-ap-southeast-2.amazonaws.com/200323_COVID19_Schools+Update+Release_www.covid19.gov.ck.pdf>.

103. Office of Te Beretitenti. @ob.gov.ki. Kiribati Declares State of Public Emergency and Partial Lock Down in Response to Corona Virus Pandemic 3:14 am, 28 March 2020. Available from: <https://www.facebook.com/ob.gov.ki/posts/1103726013322941?__tn__=K-R>.

104. School closure update [press release]. Marshall Islands: Republic of the Marshall Islands Public School System, 26 March 2020. Available from: <https://www.facebook.com/PresidentOfficeRMI/posts/2553110908295305>.

105. Ministry of Education Extends School Closure; Schools to Resume on April 20, 2020: Republic of Palau National Government; 2020 [updated 31 March 2020; cited 2020 4 April]. Available from: <https://www.palaugov.pw/ministry-of-education-extends-school-closure-schools-to-resume-on-april-20-2020/>.

106. Tonga Police. @TongaPolice. Schools in Tonga will close down. 11:49 PM, 23 March 2020. Available from: <https://twitter.com/TongaPolice/status/1242297541065994240>.

107. Declaration of Holiday for Schools in Port Vila and Luganville and SHEFA, SANMA, and TAFEA Provinces Stat. 32 (21 March 2020, 2020). Available from: <https://www.facebook.com/1674266679566197/photos/a.2102653313394196/2613569558969233/?type=3&theater>.

108. Directions for the prevention of the spread of COVID-19 - G.N. No. 128 of 2020. Government Gazette Extraordinary. 2020 20 March 2020. Available from: <https://twitter.com/BWGovernment/status/1241366896458555393>.

109. The Ministry of Information and Communication. @mic.gov.sl. Ministry of Information and Communications COVID- 19 SAFETY MEASURES. 9:30 AM, 2 April 2020. Available from: <https://www.facebook.com/229960890925264/posts/603536630234353/?d=n>.

110. Only food, fruit and vege stalls to be open at Punanganui Market from this Saturday [press release]. Cook Islands: Cook Islands Ministry of Health 25 March 2020. Available from: <http://cit-covid19.s3-ap-southeast-2.amazonaws.com/Media+release++-+Partial+closure+of+Punanganui+FINAL.PDF>.

111. National Disaster Management office Vanuatu. @ndmo.gov.vu. New directions. 8:50 PM, 1 April 2020. Available from: <https://www.facebook.com/ndmo.gov.vu/posts/1306480043074345>.

112. Botswana Government. @BWGovernment. Govt inject two billion... 12:18 PM, 27 March 2020. Available from: <https://twitter.com/BWGovernment/status/1243573146709569538>.

113. Burundi Ministry of Public Health. @mspls_bdi. With the support of... 9:05 AM, 31 January 2020. Available from: <https://twitter.com/mspls_bdi/status/1223245929278590976>.

114. Sierra Leone to Receive $7.5 Million for COVID-19 Response [press release]. The World Bank, 2 April 2020. Available from: <https://www.worldbank.org/en/news/press-release/2020/04/02/sierra-leone-to-receive-75-million-for-covid-19-response>.

115. The United States Is Leading the Humanitarian and Health Assistance Response to COVID-19 [Web page]. U.S. Department of State; 2020 [updated 27 March 2020; cited 2020 8 April]. Available from: <https://www.state.gov/the-united-states-is-leading-the-humanitarian-and-health-assistance-response-to-covid-19/>.

116. Interior Provides $858,924 to Procure GeneXpert COVID-19 Testing Kits and Machine for the U.S. Pacific Territories and Freely Associated States [Web page]. U.S. Department of the Interior; 2020 [updated 26 March 2020; cited 2020 20 April]. Available from: <https://www.doi.gov/oia/press/interior-provides-858924-procure-genexpert-covid-19-testing-kits-and-machine-us-pacific?fbclid=IwAR0qLqRVG1ZKWalNL53rh9LEHIKGx1CPmh7uDKdEuuTV8ilgA_yYToyGe-U>.

117. Statement from the President: Regarding the Nation’s Efforts in Addressing COVID-19, an Unavoidable Public Health Emergency [Web page]. Palau National Government; 2020 [updated 17 March 2020; cited 2020 20 April]. Available from: <https://www.palaugov.pw/statement-from-the-president-regarding-the-nations-efforts-in-addressing-covid-19-an-unavoidable-public-health-emergency/>.

118. World Bank provides US$5.1m for Samoa COVID-19 response [Web page]. The World Bank; 2020 [updated 27 March 2020; cited 2020 20 April]. Available from: <https://www.worldbank.org/en/news/press-release/2020/03/27/world-bank-provides-us5-1m-for-samoa-covid-19-response>.

119. China Joins Battle to Prevent COVID-19 from Solomon Islands [Web page]. Solomon Islands Government; 2020 [updated 1 April 2020; cited 2020 21 April]. Available from: <https://solomons.gov.sb/china-joins-battle-to-prevent-covid-19-from-solomon-islands/>.

120. Coronavirus disease 2019 Situation Report 9 Ministry of Health Vanuatu; 2020 1 April. Available from: <https://covid19.gov.vu/images/Situation-reports/Vanuatu_COVID19_SitRep9_01042020_1.pdf>.

121. Health Professionals Prepare for COVID-19 [Online]. Union of the Comoros; 2020 [updated 28 March 2020; cited 2020 9 April]. Available from: <https://stopcoronavirus.km/actualit%C3%A9s/2020/03/28/les-professionnels-de-la-sant%C3%A9-se-pr%C3%A9parent-pour-le-covid-19/>.

122. Lesotho Beefs Up COVID-19 Preparedness Strategies [press release]. Government of Lesotho, 10 March 2020. Available from: <https://www.gov.ls/lesotho-beefs-up-covid-19-preparedness-strategies/>.

123. WHO South Sudan. @WHOSouthSudan. To increase awareness... 9:26 AM, 30 March. Available from: <https://twitter.com/WHOSouthSudan/status/1244617038074728452>.

124. The National Center for Health and Population Education and Media - Yemen. @health.ed.yemen1. Launch of training... 10:57 AM, 22 March 2020. Available from: <https://www.facebook.com/585383058147888/posts/3053672517985584/?d=n>.

125. COVID-19 (Coronavirus) RMI Situation Report - 12. Facebook: RMI Ministry of Health and Human Services; 2020 25 March. Contract No.: 12. Available from: <https://www.facebook.com/rmimoh/photos/a.454567834705601/1524782131017494/?type=3&theater>.

126. Botswana Government. @BWGovernment. The medical supplies... 4:54 AM, 27 March 2020. Available from: <https://twitter.com/BWGovernment/status/1243461371267821568>.

127. President invites structures fighting Coronavirus to measure impact of national response [press release]. Union of The Comoros Presidency of the Republic, 22 March 2020. Available from: <https://beit-salam.km/actualit%C3%A9s/le-pr%C3%A9sident-invite-les-structures-de-lutte-contre-le-coronavirus-pour-mesurer-l%E2%80%99impact-de-la-r%C3%A9ponse-nationale.html>.

128. Malawi Government. @malawigovernment. China donates medical... 11:29 AM, 31 March 2020. Available from: <https://www.facebook.com/195859813933854/posts/1435689593284197/?d=n.%20Accessed%20on%20April%207%2C%202020>.

129. Malawi Government. @malawigovernment. Pictorial. 3:19 PM, 25 March 2020. Available from: <https://www.facebook.com/195859813933854/posts/1430629360456887/?d=n>.

130. Ministry of Health - São Tomé and Príncipe. @MSaudeSTeP. Arrived at the beginning... 2:24 PM, 1 April 2020. Available from: <https://www.facebook.com/824921004294668/posts/2804751342978281/?d=n>.

131. Ministry of Health - São Tomé and Príncipe. @MSaudeSTeP. Last Saturday... 2:32 PM, 1 April 2020. Available from: <https://www.facebook.com/824921004294668/posts/2804765256310223/?vh=e&d=n>.

132. WHO EMRO. @WHOEMRO. Special thanks to... 5:07 AM, 23 March 2020. Available from: <https://twitter.com/WHOEMRO/status/1242014998723022853>.

133. The Rodong Sinmun. Intensifying Anti-epidemic Work without Slackening Vigilance: The Rodong Sinmun; 2020 [updated 4 March 2020; cited 2020 7 April]. Available from: <http://rodong.rep.kp/en/index.php?strPageID=SF01_02_01&newsID=2020-03-04-0004>.

134. WHO helps Samoa and Tokelau to prepare for COVID-19 [Web page]. World Health Organization; 2020 [updated 9 March 2020. Available from: <https://www.who.int/samoa/news/detail/09-03-2020-who-helps-samoa-and-tokelau-to-prepare-for-covid-19>.

135. Update on the Novel Coronavirus [Web page]. Government of the Kingdom of Tonga; 2020 [updated 21 February 2020; cited 2020 21 April]. Available from: <http://www.gov.to/press-release/update-on-the-novel-coronavirus/>.

136. Press Conference on the Coronavirus Epidemic [press release]. Ministry of Public Health and the Fight to Control AIDS, 1 February 2020. Available from: <http://minisante.bi/?p=540>.

137. Beit-Salam - Presidency of the Union of the Comoros. @beitsalam. A marathon day... 4:10 PM, 25 March 2020. Available from: <https://www.facebook.com/beitsalam/posts/2557025161239325>.

138. DTM South Sudan - COVID-19 Preparedness Mobility Update 1 (23-29 March 2020). United Nations International Organization for Migration; 2020 1 April. Report No.: 1 Contract No.: 1. Available from: <https://migration.iom.int/reports/dtm-south-sudan-%E2%80%94-covid-19-preparedness-mobility-update-1-23-29-march-2020>.

139. Marufjon Hojiboev: “No Cases Of Coronavirus Infection Have Been Registered In The Province So Far” [press release]. Ministry of Health and Social Protection of the Republic of Tajikistan, 6 April 2020. Available from: <http://moh.tj/%d0%bc%d1%83%d0%be%d0%b2%d0%b8%d0%bd%d0%b8-%d1%81%d0%b0%d1%80%d0%b4%d0%be%d1%80%d0%b8-%d1%80%d0%b0%d1%91%d1%81%d0%b0%d1%82%d0%b8-%d1%82%d0%b0%d0%bd%d0%b4%d1%83%d1%80%d1%83%d1%81%d1%82%d3%a3-%d0%b4/>.

140. Constitutional Emergency Order No. 20-01, 20-01 (2020). Available from: <https://www.fsmgov.org/fsmun/pubhealt0.pdf>.
